# Sunning themselves in heaps, knots, and snarls: The extraordinary abundance and demography of island watersnakes

**DOI:** 10.1002/ece3.4191

**Published:** 2018-07-04

**Authors:** Richard B. King, Kristin M. Stanford, Peter C. Jones

**Affiliations:** ^1^ Department of Biological Sciences Northern Illinois University DeKalb Illinois; ^2^ Institute for the Study of the Environment, Sustainability and Energy Northern Illinois University DeKalb Illinois; ^3^ F. T. Stone Laboratory Ohio State University Put‐in‐Bay Ohio

**Keywords:** body size, capture–mark–recapture, life history, population estimation, process variance, realized population growth, survival, vital rates

## Abstract

Snakes represent a sizable fraction of vertebrate biodiversity, but until recently, data on their demography have been sparse. Consequently, generalizations regarding patterns of variation are weak and the potential for population projections is limited. We address this information gap through an analysis of spatial and temporal variation in demography (population size, annual survival, and realized population growth) of the Lake Erie Watersnake, *Nerodia sipedon insularum*, and a review of snake survival more generally. Our study spans a period during which the Lake Erie Watersnake was listed as threatened under the U.S. Endangered Species Act, recovered, and was delisted. We collected capture–mark–recapture data at 14 study sites over 20 years, accruing 20,000 captures of 13,800 individually marked adults. Lake Erie Watersnakes achieve extraordinary abundance, averaging 520 adults per km of shoreline (ca. 260 adult per ha) at our study sites (range = 160–1,600 adults per km; ca. 80–800 adults per ha) and surpassing population recovery and postdelisting monitoring criteria. Annual survival averages 0.68 among adult females and 0.76 among adult males, varies among sites, and is positively correlated with body size among study sites. Temporal process variance in annual survival is low, averaging 0.0011 or less than 4% of total variance; thus, stochasticity in annual survival may be of minor significance to snake extinction risk. Estimates of realized population growth indicate that population size has been stable or increasing over the course of our study. More generally, snake annual survival overlaps broadly across continents, climate zones, families, subfamilies, reproductive modes, body size categories, maturation categories, and parity categories. Differences in survival in relation to size, parity, and maturation are in the directions predicted by life history theory but are of small magnitude with much variation around median values. Overall, annual survival appears to be quite plastic, varying with food availability, habitat quality, and other ecological variables.

## 1 INTRODUCTION

The availability of accurate demographic parameter estimates is central to understanding population dynamics (Caswell, 2001), evaluating life history evolution (Roff, 1992; Stearns, 1992), and modeling extinction risk (Akçakaya, 2004; Lacy, 1993, 2000). Generating such estimates requires long‐term monitoring data, often of marked individuals. Historically, game species and species of economic importance have been the targets of such monitoring, but increasingly, efforts have broadened to include species of conservation concern. At the same time, analytical methods for generating demographic parameter estimates have improved and increased in sophistication (Halstead, Wylie, Coates, Valcarcel, & Casazza, 2012; White & Burnham, 1999). Consequently, demographic parameter estimates are becoming available for a widening range of taxa (Mesquita et al., 2015; Salguero‐Gómez et al., 2015, 2016), offering the possibility of improved interpretation and generalization, including evaluations of *r*‐*K*, slow‐fast, and pace‐of‐life syndromes (Bielby et al., 2007; Dunham, Miles, & Reznick, 1988; Gaillard et al., 1989, 2005; Gangloff et al., 2017; Hille & Cooper, 2015; Réale et al., 2010; Ricklefs & Wikelski, 2002; Wiersma, Muñoz‐Garcia, Walker, & Williams, 2007) and niche classification (Pianka, Vitt, Pelegrin, Fitzgerald, & Winemiller, 2017; Winemiller, Fitzgerald, Bower, & Pianka, 2015).

Snakes represent a sizable fraction of vertebrate biodiversity; their 3,400 species constitute 10% of extant tetrapods and 35% of extant squamates (lizards and snakes; http://www.reptile-database.org/db-info/SpeciesStat.html). Although often secretive and infrequently encountered, snakes can be abundant and, as tertiary predators, exert top‐down influences on ecosystem function (Jones, King, Stanford, Lawson, & Thomas, 2009; Willson & Winne, 2016 and citations therein). Despite their diversity, snakes are poorly represented among life history data compilations, comprising, for example, just four entries among the 1,927 matrix population models in the COMADRE Animal Matrix Database (Salguero‐Gómez et al., 2016; http://www.compadre-db.org/ accessed 19 October 2017).

Here, we provide a case study of the demography of the Lake Erie Watersnake, *Nerodia sipedon insularum* (Conant & Clay). Using data from 14 study sites collected over 20 years, we estimate population size, annual adult survival, and its variance and realized population growth. We characterize the degree to which annual survival and realized population growth vary among sexes, sites, and years. Sex effects are likely to arise from differences in body size and reproductive allocation between males and females. Females exceed males in length and mass, possibly making them less vulnerable to predation. Males engage in active mate‐searching behaviors in spring, and females frequently bask, while gestating during summer, both potentially risky behaviors; females also produce large litters of energetically expensive offspring. Site and year effects are likely to arise from spatial and temporal variation in biotic (predator and prey abundance) and abiotic (weather and microclimate) factors. Thus, our analyses provide insight into the degree to which adult survival is influenced by year‐to‐year and local site‐to‐site variation in the environment.

We place the variation we see in annual adult survival of Lake Erie Watersnakes in context by reviewing other studies of snake survival that have utilized contemporary estimation techniques. Our review updates a summary compiled 30 years ago (tables 9‐5 in Parker & Plummer, 1987) that relied heavily on survival estimates from return rates of marked animals (e.g., to overwintering dens) and from life tables generated from inferred age structure; methods that do not account for imperfect and varying detection probability. Following Parker and Plummer (1987), we consider variation in annual adult survival among families, subfamilies, continents, and climate zones and its relationship to reproductive mode, body size, age at maturity, and reproductive frequency.

## 2 MATERIALS AND METHODS

### 2.1 Study species

**Figure 1:**
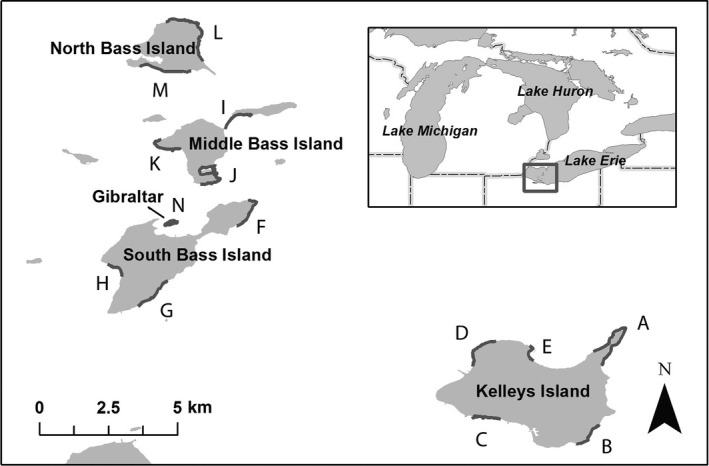
The island region of western Lake Erie showing 14 sites included in this study. Sites, include on Kelleys Island—Long Point (A), Southeast Shore (B), South Shore (C), Minshall (D), and State Park (E); on South Bass Island—East Point (F), East Shore (G), and State Park (H); on Middle Bass Island—East Point (I), State Park (J), and West End (K); on North Bass Island—N,NE,E Shore (L) and South Shore (M); and on Gibraltar Island in its entirety (N)

The Lake Erie Watersnake, *Nerodia sipedon insularum*, is endemic to the islands of western Lake Erie (North America; Figure 1). It differs in color pattern from mainland Northern Watersnakes (*N. s. sipedon*, Linnaeus) due to a dynamic balance between natural selection favoring less patterned individuals along rocky island shorelines and gene flow from nearby mainland populations (King & Lawson, 1997). Lake Erie Watersnakes consume amphibians and fish, including the invasive Round Goby (*Neogobius melanostomus*), in the nearshore waters of Lake Erie (Jones et al., 2009; King, Ray, & Stanford, 2006; King, Stanford, & Ray, 2008). They utilize shoreline retreats and basking sites during the active season and both shoreline and inland hibernation sites during winter (Stanford, King, & Wynn, 2010). Like other New World natricines, Lake Erie Watersnakes are viviparous, and adult females produce large litters (mean litter size = 26, King et al., 2008) of independent young annually.

Restricted geographic distribution and declining population size led to listing of the Lake Erie Watersnake as threatened under the U.S. Endangered Species Act in fall 1999 (U.S. Fish and Wildlife Service, 1999). Recovery efforts focused on population monitoring, habitat management, and a reduction in human‐caused mortality (U.S. Fish and Wildlife Service, 2003). These efforts occurred simultaneously with an exponentially growing Round Goby population (Johnson, Allen, Corkum, & Lee, 2005) and a shift in watersnake diet to this new and abundant food source (Jones et al., 2009; King, Ray, et al., 2006; King et al., 2008). Successful achievement of recovery criteria resulted in the delisting of the Lake Erie Watersnake in summer 2011 (U.S. Fish and Wildlife Service, 2011). As required under the Endangered Species Act, postdelisting monitoring was implemented to ensure that the Lake Erie Watersnake sustained itself following delisting (U.S. Fish and Wildlife Service, 2011). Population monitoring prior to listing, during recovery, and postdelisting provides an extensive database for the demographic analyses presented here.

### 2.2 Field protocols

Data analyzed here were collected from 1996 through 2015 at study sites on five U. S. islands in western Lake Erie (Figure 1). Data collection accelerated in 2001 when a program of intensive annual population censuses was initiated. These censuses spanned a period of about 2 weeks (late May to mid‐June) each spring. Some sites were also sampled outside of this period, either to provide more accurate population estimates or to meet other research objectives. As of 2005, 14 intensive study sites were included (Figure 1), encompassing 0.7–2.8 km of shoreline each and accounting for about 30% (17.9 km) of the total shoreline (57.7 km) of these islands. The number of study sites was reduced to 13 in 2011 and 12 in 2012 when two sites with inconsistent and low capture rates were dropped.

Censuses consisted of area‐constrained searches of suitable shoreline habitat. Watersnakes were captured by hand, classified by sex, measured to obtain snout‐vent length (SVL), and weighed. Watersnakes were permanently marked using passive integrated transponders (PIT tags) injected under the skin. Following marking, snakes were released at their site of capture. Watersnakes were also temporarily marked using a latex paint stick (All‐Weather Paintstik^®^ Livestock Marker, LA‐CO Industries, Inc.). Temporary marks alerted field workers that a snake had been recently processed and that it needs only be scanned before release. Watersnakes were classified as adults if SVL ≥ 430 mm (males) or ≥590 mm (females) (King, 1986; King, Queral‐Regil, & Stanford, 2006; King, Stanford, Jones, & Bekker, 2016). Processing was carried out in the field, at the F. T. Stone Laboratory, our research base during censuses of sites on South Bass, Middle Bass, North Bass and Gibraltar island, or at “South Bay,” a rental property used as our research base during censuses of sites on Kelleys Island. Less intensive censuses occurred at additional study sites periodically throughout the study (King, Queral‐Regil, et al., 2006). Results from those sites are included here only as they pertain to documenting movements of animals among study sites. Census participants included federal and state agency staff, university faculty and students, zoo staff, naturalists, and area residents working in teams of ca. 3–8 individuals. Correctly identifying marked animals was a primary goal of snake processing, and errors resulting from tag loss, tag failure, detection failure, scanner failure, or observer error were minimal (more details are provided in Supporting Information Data S1).

### 2.3 Population size

Adult Lake Erie Watersnake population sizes were estimated using the Jolly–Seber model (Jolly, 1965; Seber, 1965) via the program JOLLY (http://www.mbr-pwr.usgs.gov/software.html) with 95% confidence intervals calculated using Manly’s method (Krebs, 1998; Manly, 1984). Capture histories were created for each snake using 0’s to denote years in which a given snake was not captured, 1’s to denote years in which a snake was captured, and 2’s to denote snakes that were found dead or that were released unmarked. Multiple captures of the same animal within a year were treated as a single capture for the purposes of population estimation. Because capture probabilities differed between sexes (Results), estimates were computed separately for males and females. Population estimates and confidence intervals were rounded to the nearest 10. Population density was computed by dividing population estimates by the linear extent of shoreline sampled within each study site (King, Queral‐Regil, et al., 2006).

The Jolly–Seber model is an open population model, allowing for recruitment, death, immigration, and emigration between sampling occasions (Jolly, 1965; Seber, 1965). However, emigration is assumed to be permanent, and thus, animals that are captured at one study site were treated as new individuals if they are later recaptured at another study site. To assess the extent of movement among study sites and the degree to which emigration was permanent, we tallied cases of recaptures that occurred at a site different from the site of prior capture.

### 2.4 Annual survival

We estimated adult survival (*ϕ*) and recapture (*p*) parameters for Lake Erie Watersnakes using the Cormack–Jolly–Seber (CJS) model in RMark version 2.1.7 (Laake, 2013) and Program MARK version 8.0 (White & Burnham, 1999). We used R version 3.2.2 (R Core Team, 2015) to construct and run all models. For sites where sampling began after 2000 or ended prior to 2015, recapture parameters were fixed to zero by deleting the model’s design data for years when no sampling occurred. We used the fully parameterized model *ϕ*(sex*site*time)*p*(site*time*sex) as our global model. Goodness of fit was assessed for the global model in U‐CARE (Choquet, Lebreton, Gimenez, Reboulet, & Pradel, 2009). We calculated ĉ for the global model to check for overdispersion. We anticipated an interactive effect of site and time on recapture probabilities based on site‐to‐site and year‐to‐year variation in capture success arising from constraints imposed by weather and sampling logistics. Therefore, in addition to the global model, we evaluated candidate models that included *p*(site*time), *p*(site*time+sex), and *p*(site*time*sex) and additive and interactive effects of site, time, and sex on *ϕ* (Supporting Information Table S1). We excluded several highly parameterized models with *ϕ*(sex*site+time) and *ϕ*(sex+site*time) because of data sparseness and the likelihood of inestimable parameters.

### 2.5 Process variance in annual survival

Temporal process variance is the portion of the total variance that is attributable to the actual variation in a demographic parameter over time and can be estimated via a variance components approach as total variance minus sampling error (Gould & Nichols, 1998). Similarly, spatial process variance is the portion of the total variance that is attributable to actual variation among sites. We used Program MARK version 8.0 (White & Burnham, 1999) to estimate the temporal process variance in survival from the highest ranked model that included time effects on *ϕ* and spatial process variance in survival from the highest ranked model that included site effects on *ϕ*.

### 2.6 Realized population growth

We estimated realized population growth (*λ*) using the Pradel *ϕ* and *λ* parameterization within Program MARK. We used our top‐ranked CJS model, *ϕ*(sex*site)*p*(site*time+sex) (cf. Cam, 2012; Anthony et al., 2006; Seamans & Gutierrez, 2007; Mullin, Colwell, McAllister, & Dinsmore, 2010; Korfanta, Newmark, & Kauffman, 2012), and then considered additive and interactive effects of site, sex, and time on *λ* (Supporting Information Table S2). Because sampling was initiated in different years among sites, candidate Pradel models all included *λ*(site) in their parameterization to avoid the effects of a changing study area (Cooch & White, 2017; Pradel, 1996).

### 2.7 Associations between demography and body size among sites

Watersnakes continue to grow after reaching reproductive maturity and so may achieve greater body size at sites where survival is high. However, there is also much individual variation in the asymptotic size of Lake Erie Watersnakes (King et al., 2016) so larger individuals are not necessarily older. Consequently, it is unclear whether body size might show an association with survival, and if it does, whether high survival promotes increased body size, large body size promotes increased survival (e.g., by making snakes difficult for predators to subdue; King, 1993), or some other mechanism underlies variation in both. To determine if any such association exists, we computed the mean SVL of the largest 10% of individuals of each sex captured at each site. We then used one‐tailed Pearson’s correlation with test for positive associations between SVL and *ϕ* separately for males and females.

### 2.8 Review of snake survival

We collated estimates of survival that utilized maximum likelihood‐based methodologies (including known‐fate analyses) to analyze capture–mark–recapture data while accounting for imperfect detection. We categorized species by family, subfamily, continent, climate zone of study location (temperate, >40°N or S; subtropical, 23.5–40°N or S; tropical, <23.5°N or S), and reproductive mode (oviparous and viviparous). In addition, we classified species by body size as small (lower adult size limit from 150 to 350 mm SVL and upper adult size limit from 250 to 800 mm SVL), medium (400–500 and 600–1,200 mm SVL), or large (600–1,400 and 1,000–3,000 mm SVL); by attainment of reproductive maturity as early (3 years or less), intermediate (3–4 years), or late (more than 4 years); and by parity as reproducing on an annual or shorter interval vs. biennial or longer interval.

## 3 RESULTS

From 1996 to 2015, a total of 13,802 individual adult Lake Erie Watersnakes were captured 20,025 times at 14 intensive study sites (Supporting Information Table S3). Included among these were 129 animals that had been marked in 1 year and found dead in a subsequent year, 117 unmarked individuals found dead, and 27 live individuals that were released unmarked. Excluding dead and unmarked animals, the number of individual adult Lake Erie Watersnakes ranged from 284 to 1,854, and the number of captures ranged from 330 to 2,694 per study site (Supporting Information Table S3).

Most snakes were captured in just a single year, but some individuals were recaptured in as many as 8 or 9 years (Supporting Information Table S4). Of animals captured in more than 1 year, most were captured in two successive years but gaps of 1 or more years between captures were common (Supporting Information Table S4). At the extreme were two animals first captured in 2002 and subsequently in 2014 (a gap of 11 years) and 2015 (a gap of 12 years). The time span between first and last capture ranged up to 15 years (Supporting Information Table S4).

### 3.1 Extent and permanence of emigration

We identified 57 cases of Lake Erie Watersnakes that moved among our 14 study sites (more details are provided in Supporting Information Data S2, Table S5, Figure S1). In contrast, 9,358 recaptures that occurred at the same site as prior capture. Thus, only 0.6% of recaptures involved moves among sites.

### 3.2 Population size

Capture–mark–recapture data allowed use of the Jolly–Seber method to estimate adult male and female population size at most sites in most years from 2001 to 2014 (Table 1, Supporting Information Table S6, Figure S2). Combining males and females and averaging across years, estimated population size varied among study sites from fewer than 200 (Kelleys Island Minshall, Kelleys Island State Park) to more than 1,000 adults (Kelleys Island South Shore; Table 1, Supporting Information Table S6, Figure S2). Density estimates were also highly variable, ranging from 100 to 1,600 adults per km (Table 1). In general, 95% confidence limits of population estimates were broad with lower and upper limits frequently differing by a factor of two or more (Supporting Information Table S6, Figure S2). However, estimates were often quite consistent among years, sometimes varying by just a few 10 s of individuals (e.g., South Bass Island State Park, Kelleys Island State Park, and Gibraltar Island; Table 1, Supporting Information Table S6, Figure S2). Males typically had a higher population estimate than females at a given site; however, male and female confidence intervals overlapped broadly (Table 1, Supporting Information Table S6, Figure S2).

**Table 1:** Population size and density, annual adult survival (*ϕ*) and associated temporal process variance, realized population growth (*λ*), and maximum body size of adult female and male Lake Erie Watersnakes at 14 study sites

Site (length, km)	Sex	Population size and density		Annual adult survival		Realized population growth	Maximum body size (mm)	
		Adults	Adults per km	*ϕ* (CI)	Process variance	*λ* (CI)	*n*	SVL (Range)
KI Long Point (2.8)	Female	230	160	0.85 (0.79–0.89)	<0.0001	**1.06** (1.01–1.11)	28	1,046 (1,034–1,057)
	Male	210		0.84 (0.77–0.89)	–	**1.11** (1.06–1.17)	27	793 (783–802)
KI SE Shore (1.0)	Female	270	650	0.70 (0.67–0.73)	0.0006	1.01 (0.98–1.03)	82	981 (976–987)
	Male	380		0.79 (0.76–0.81)	0.0004	1.02 (1.00–1.04)	103	769 (764–774)
KI South Shore (0.7)	Female	270	1,600	0.55 (0.49–0.61)	0.0006	0.97 (0.94–1.00)	54	965 (956–975)
	Male	850		0.81 (0.78–0.83)	0.0002	**1.04** (1.02–1.07)	142	760 (756–764)
KI Minshall (1.3)	Female	50	100	0.51 (0.36–0.65)	0.0080	0.96 (0.85–1.08)	11	969 (952–986)
	Male	80		0.71 (0.59–0.80)	0.0001	0.95 (0.85–1.06)	16	750 (738–762)
KI State Park (0.7)	Female	60	210	0.63 (0.58–0.68)	0.0005	**1.05** (1.02–1.08)	31	1,002 (991–1,013)
	Male	90		0.72 (0.68–0.76)	0.0004	**1.04** (1.01–1.07)	33	767 (762–773)
SBI East Point (1.3)	Female	230	330	0.71 (0.66–0.76)	0.0079	**1.09** (1.04–1.15)	51	991 (983–1,000)
	Male	200		0.74 (0.70–0.78)	0.0007	1.00 (0.95–1.05)	57	783 (776–790)
SBI East Shore (1.0)	Female	330	780	0.71 (0.67–0.76)	0.0006	1.00 (0.97–1.04)	54	1,010 (1,002–1,017)
	Male	450		0.85 (0.82–0.87)	0.0002	0.98 (0.95–1.01)	78	785 (780–789)
SBI State Park (0.7)	Female	170	570	0.71 (0.68–0.73)	0.0006	1.01 (0.99–1.03)	102	930 (923–937)
	Male	230		0.71 (0.69–0.74)	0.0006	0.96 (0.94–0.98)	104	719 (713–725)
MBI East Point (1.0)	Female	250	810	0.64 (0.56–0.71)	0.0009	0.94 (0.89–0.99)	32	995 (983–1,007)
	Male	560		0.83 (0.78–0.87)	0.0003	0.96 (0.91–1.00)	63	756 (750–761)
MBI State Park (1.0)	Female	200	450	0.65 (0.61–0.69)	0.0007	1.00 (0.97–1.04)	64	1,006 (999–1,013)
	Male	250		0.67 (0.62–0.71)	0.0007	0.99 (0.95–1.03)	59	772 (765–779)
MBI West End (1.1)	Female	540	910	0.74 (0.64–0.82)	0.0003	1.07 (1.00–1.15)	48	1,062 (1,052–1,071)
	Male	460		0.92 (0.81–0.97)	–	**1.12** (1.05–1.19)	64	786 (779–793)
NBI NE,E,SE Shore (2.4)	Female	180	180	0.70 (0.64–0.75)	0.0008	1.05 (1.00–1.10)	44	970 (958–982)
	Male	240		0.66 (0.59–0.72)	0.0009	1.02 (0.96–1.07)	43	727 (714–740)
NBI South Shore (2.0)	Female	240	270	0.72 (0.69–0.75)	0.0006	**1.11** (1.09–1.14)	122	993 (986–1,000)
	Male	300		0.67 (0.63–0.72)	0.0006	**1.14** (1.11–1.17)	106	746 (739–753)
Gibraltar (0.9)	Female	70	260	0.74 (0.69–0.78)	0.0005	1.04 (1.00–1.08)	45	1,014 (1,003–1,024)
	Male	160		0.73 (0.69–0.77)	0.0005	1.02 (0.98–1.06)	49	772 (764–779)

Note

To remove effects of year, population size was averaged and *λ* was estimated from *ϕ*(sex*site)*p*(site*time+sex)*λ*(site*sex). Dashes indicate unestimable values. Estimates of *λ* that are significantly greater than 1.0 are indicated in bold; CI refers to 95% confidence interval; *n* refers to the number of animals constituting the largest 10%.

### 3.3 Annual survival

An overall goodness of fit test of the global model was nonsignificant (*p* = 0.91), indicating a good fit of the data to CJS open population models. The resultant estimate for ĉ was 0.87, indicating an absence of overdispersion and precluding the need to adjust AICc values (Burnham & Anderson, 2002). The most parsimonious CJS model from our candidate set, *ϕ*(sex*site)*p*(site*time+sex), included interactive effects of sex and site on survival and interactive effects of site and time and an additive effect of sex on recapture probability (Supporting Information Table S1). This model had weight = 0.76. The second highest ranked model, *ϕ*(sex*site+time)*p*(site*time+sex) (weight = 0.24), differed only in that it included an additive effect of time on survival.

Recapture probability was highest at South Bass Island (SBI) State Park, exceeding 0.25 in all years and frequently exceeding 0.5 (Supporting Information Figure S3, Table S7). In contrast, recapture probability was consistently low at KI South Shore, SBI East Shore, MBI East Point, and MBI West End, falling below 0.25 in nearly all years. Recapture probabilities were frequently between 0.25 and 0.5 at other sites. Recapture probabilities were 3%–5% higher among females than males.

**Figure 2:**
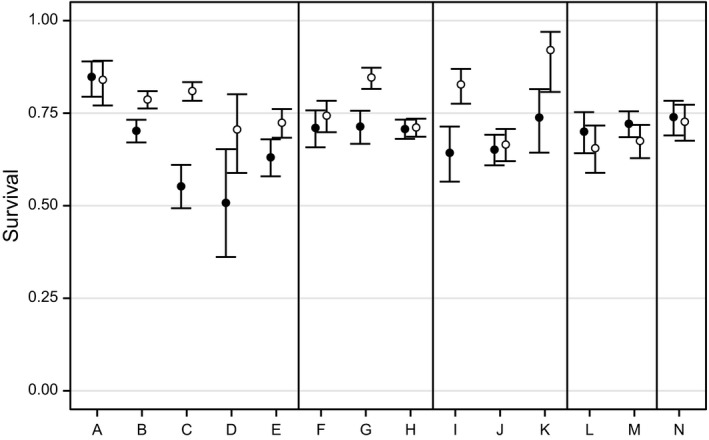
Estimated annual adult survival, *ϕ*, of male (unfilled circles) and female (filled circles) Lake Erie Watersnakes and associated 95% confidence intervals. Letters identify study sites as in Figure 1, and vertical lines separate islands

Overall, annual adult male survival averaged 0.76 and annual adult female survival averaged 0.68 (Figure 2, Table 1). However, the difference in survival between males and females varied among sites. Male survival was approximately equal to female survival at some sites (KI Long Point, SBI East Point, SBI State Park, MBI State Park, NBI NE,E,SE Shore, NBI South Shore, and Gibraltar) but exceeded female survival, by 0.09–0.26, at other sites (KI Southeast Shore, KI South Shore, KI Minshall, KI State Park, SBI East Shore, MBI East Point, and MBI West End; Figure 2, Table 1). Confidence intervals for sex‐ and site‐specific estimates averaged 0.10 (range = 0.05–0.29; Table 1).

### 3.4 Process variance in annual survival

Temporal process variance in annual adult survival could be estimated for 26 of 28 sex–site combinations (Table 1) and was generally low, averaging just 0.0011 (range = <0.0001 to 0.0080 among sites; Table 1). In contrast, total variance in adult survival over time averaged 0.0264 (range = 0.0132‐0.0773 among sites). Thus, temporal process variance averaged less than 4% of total variance (range = <0.1%–30.2% among sites). Spatial process variance in annual adult survival was 0.005 for both males and females (24% of total variance).

### 3.5 Realized population growth

**Figure 3:**
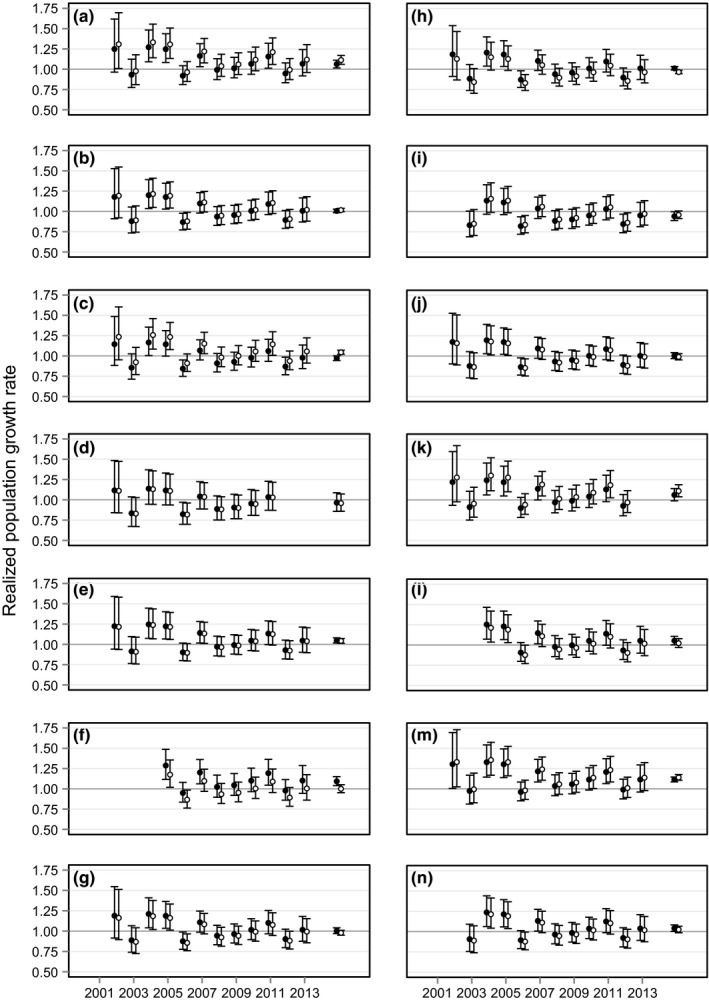
Estimated annual adult realized population growth, *λ*, of male (unfilled circles) and female (filled circles) Lake Erie Watersnakes and associated 95% confidence intervals. Study sites, identified by letters as in Figure 1, are represented by separate panels. Points on the far right of each panel represent mean realized population growth across years estimated using the model *ϕ*(sex*site)*p*(site*time+sex)*λ*(site*sex)

**Figure 4:**
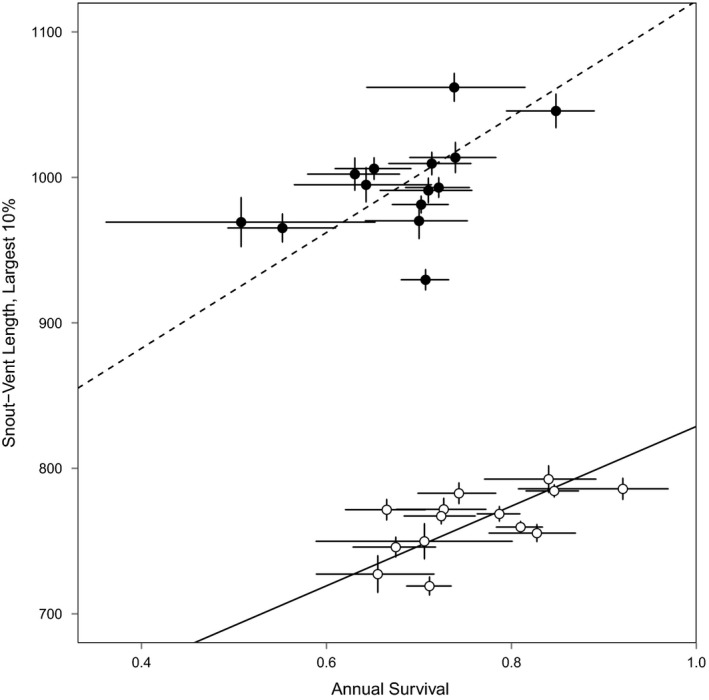
Association between estimated annual adult survival and body size as indicated by the mean SVL of the largest 10% of animals captured from 2000 to 2015. Points represent the 14 study sites included in this analysis. Females are represented by the upper cluster of points; males are represented by the lower cluster of points. Whiskers represent 95% confidence intervals. Lines represent reduced major axis regressions for females (dashed) and males (solid)

The most parsimonious Pradel model from our candidate set, *ϕ*(sex*site)*p*(site*time+sex)*λ*(site*sex+time), included interactive effects of site and sex and an additive effect of time on realized population growth (Supporting Information Table S2). This model had weight = 1.00, indicating its clear precedence over other candidate models. Confidence intervals for realized population growth were wide for the early years of the study but decreased over time (Figure 3). Of 28 site–sex combinations, realized population growth was significantly greater than one in nine, did not differ from one in 17, and was significantly less than one in only two. Realized population growth was similar in males and females, averaging 1.025 and 1.026, respectively (range = 0.95–1.14 and 0.94–1.11; Table 1, Figure 4, Supporting Information Table S8).

### 3.6 Associations between survival and body size among sites

Mean SVL of the largest 10% of animals captured from 2000 to 2015 varied among sites from 719 to 793 mm in males and from 930 to 1,062 mm in females (Table 1). Across sites, mean SVL was positively correlated between the sexes (*r* = 0.81, *df* = 12, *p* < 0.001). Furthermore, mean SVL was positively correlated with *ϕ* in both males (*r* = 0.61, *df* = 12, *p* = 0.010) and females (*r* = 0.52, *df* = 12, *p* = 0.030; Figure 4).

### 3.7 Review of snake survival

**Figure 5:**
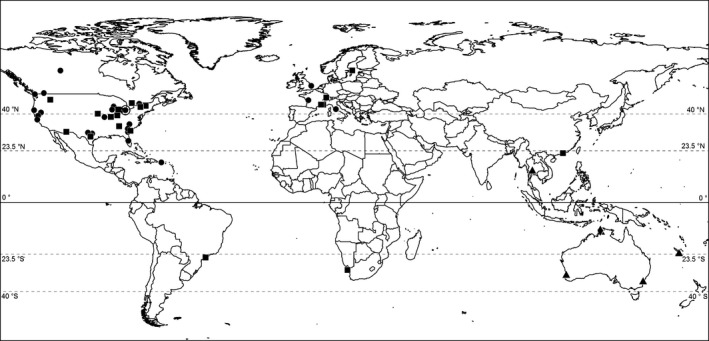
Localities of studies providing estimates of annual survival among of snakes (Colubridae—circles, Viperidae—squares, Elapidae—triangles, and Pythonidae—diamonds). The location of our case study of the Lake Erie Watersnake (north central USA) is circled. Dashed lines separate tropical (<23.5°N or S), subtropical (23.5–40°N or S), and temperate (>40°N or S) climate zones

We collated information from 65 studies of 45 species that provided estimates of survival for one or more age classes of snakes (Table 2, Supporting Information File S1, Figure 5). After averaging across sexes, years, and local populations, we identified 55 estimates (48 species) of adult annual survival, 18 estimates (16 species) of annual juvenile survival, and 12 estimates (10 species) of annual neonatal survival (Table 2). Thirteen studies provided separate estimates of adult male and adult female annual survival (Supporting Information File S1).

**Table 2:** Estimates of annual survival in snakes based on known‐fate analyses of telemetry data (study type = T) or analyses of capture–mark–recapture data that account for imperfect detection (study type = CMR)

Species	Study type	Grouping variables and covariates					Annual survival			Continent	Climate area	Reproductive mode	Size	Maturation	Parity	Source
		Year	Sex	Size, Stage, or Age	Site	Other	Adult	Juvenile	Neonate							
Colubridae, Colubrinae																
*Drymarchon couperi*	CMR	−	−	+		Season−	0.74	0.52		NA	Subt	O	L	I	A	Hyslop et al. (2012)
*Drymarchon couperi*	T		−		+	Season−	0.91			NA	Subt	O	L	I	A	Breininger et al. (2012), Hyslop et al. (2012) (maturation, parity)
*Hierophis viridiflavus*	CMR	+	−			Weather−	0.55			E	Temp	O	L	I	A	Lelievre et al. (2013), Zuffi, Fornasiero, and Bonnet (2007) (size), Fornasiero, Bonnet, Dendi, and Zuffi (2016) (maturation), Capula, Filippi, Luiselli, and Jesus (1997) (parity)
*Lampropeltis triangulum*	CMR						0.72			NA	Temp	O	M	I	A	Hileman, Kapfer, Muehlfeld, and Giovanni (2015), Fitch (1999) (maturation), other NA colubrinae (parity)
*Pantherophis obsoletus*	T	+					0.76			NA	Subt	O	L	I	A	Sperry and Weatherhead (2008, 2009) (size), Fitch (1999) (maturation), other NA colubrinae (parity)
*Pituophis catenifer*	T						0.73			NA	Temp	O	L	I	A	Williams, Hodges, and Bishop (2014), Parker and Brown (1980) (maturation in *P. melanoleucus*), Diller and Wallace (1996) (parity in *P. melanoleucus*)
*Stegonotus cucullatus*	CMR	−	−			Pre− vs. posttoad invasion−	0.57			Au	Trop	O	L	E	A	Brown et al. (2013), Dubey, Brown, Madsen, and Shine (2009) (size), Brown, Shine, and Madsen (2002) (maturation, parity)
*Zamenis longissimus*	CMR	+	−			Weather+	0.51			E	Temp	O	L	I	A	Lelievre et al. (2013), Naulleau and Bonnet (1995) (size, parity), other temperate colubrinae (maturation)
Colubridae, Dipsadinae																
*Diadophis punctatus*	CMR						0.79	0.62	0.76	NA	Temp	O	S	E	A	Riedle (2014), Ernst and Ernst (2003) (parity)
Colubridae, Natricinae																
*Natrix natrix*	CMR	−		−			0.66			E	Temp	O	L	I	A	Sewell, Baker, and Griffiths (2015), Gregory (2004) (size), Madsen (1983) (maturation), Luiselli et al. (2011) (parity)
*Natrix tessellata*	CMR	+	−	+			0.73			E	Temp	O	M	I	A	Luiselli et al. (2011), Luiselli, Capula, and Shine (1997) (parity)
*Nerodia erythrogaster*	T		+	+			0.71			NA	Temp	V	M	E	A	Roe, Attum, and Kingsbury (2013), Gibbons and Dorcas (2004) (maturation in other *Nerodia*, parity)
*Nerodia fasciata*	CMR		−				0.92			NA	Subt	V	M	E	A	Willson, Winne, and Todd (2011), Conant (1975) (size); Gibbons and Dorcas (2004) (maturation in other *Nerodia*, parity)
*Nerodia harteri*	CMR	+	−	+	−		0.23	0.14		NA	Subt	V	S	E	A	Whiting et al. (2008), Greene, Dixon, Whiting, and Mueller (1999) (size, parity), Gibbons and Dorcas (2004) (maturation)
*Nerodia sipedon*	T		+	+			0.50			NA	Temp	V	M	E	A	Roe et al. (2013), Gibbons and Dorcas (2004) (maturation, parity)
*Nerodia sipedon*	CMR	+	+		+		0.72			NA	Temp	V	M	I	A	This study, King et al. (2016) (maturation), Stanford (2012) (parity)
*Nerodia sipedon*	CMR	−	+	+		Marking+		0.53	0.18	NA	Temp	V				Stanford (2012)
*Nerodia sipedon*	CMR						0.55			NA	Temp	V	M	I	A	Brown and Weatherhead (1999), Weatherhead, Barry, Brown, and Forbes (1995) (size, parity)
*Nerodia sipedon*	CMR								0.31	NA	Temp	V				Kissner and Weatherhead (2005)
*Nerodia sipedon*	CMR							0.19		NA	Temp	V				Cecala, Price, and Dorcas (2010)
*Seminatrix pygaea*	CMR		−				0.88			NA	Subt	V	S	E	A	Willson et al. (2011), Winne, Willson, and Gibbons (2006) (size, parity), Gibbons and Dorcas (2004) (maturation)
*Thamnophis atratus*	CMR	−	+				0.60			NA	Temp	V	M	I	A	Lind, Welsh, and Tallmon (2005), Rossman, Ford, and Seigel (1996) (size), other *Thamnophis* (maturation, parity)
*Thamnophis butleri*	CMR	+				Invasive plant management−	0.48			NA	Temp	V	S	E	A	E. T. Hileman, Personal Communication
*Thamnophis elegans*	CMR	+	+	+	+		0.63	0.56	0.35	NA	Temp	V	M	I	A	Miller et al. (2011), Bronikowski and Vleck (2010) (size, maturation)
*Thamnophis elegans*	CMR	+	+	+	+		0.75	0.81	0.67	NA	Temp	V	S	L	B	Miller et al. (2011), Bronikowski and Vleck (2010) (size, maturation)
*Thamnophis gigas*	T	+		−	+	Condition−, terrestrial vs. aquatic habitat+; linear vs. areal habitat−	0.61			NA	Temp	V	M	L	A	Halstead et al. (2012), Wylie, Casazza, Gregory, and Halstead (2010) (size), Rossman et al. (1996) (maturation); other *Thamnophis* (parity)
*Thamnophis radix*	CMR		+	+			0.43	0.42	0.16	NA	Temp	V	M	E	A	Stanford and King (2004)
*Thamnophis sirtalis*	CMR						0.67			NA	Temp	V	M	E	A	Larsen and Gregory (1989), Rossman et al. (1996) (maturation, parity)
*Thamnophis sirtalis*	CMR	+					0.76			NA	Temp	V	M	E	A	Halstead et al. (2011), Rossman et al. (1996) (maturation, parity)
*Tropidonophis mairii*	CMR	−	−			Pre− vs. posttoad invasion+	0.26			Au	Trop	O	M	E	A	Brown et al. (2013), Brown and Shine (2002) (size, maturation, parity)
Colubridae, Xenodontinae																
*Borikenophis portoricensis*	CMR		+				0.50			NA	Trop	O	M			Hileman, King, et al. (2017), Hileman, Powell, et al. (2017)
*Contia tenuis*	CMR	−	−	+			0.75	0.66		NA	Temp	O	S	L	B	Govindarajulu, Isaac, Engelstoft, and Ovaska (2011)
Elapidae, Elapinae																
*Naja kaouthia*	CMR			+			0.93	0.48		As	Trop	O	L	I		Chaitae (2011)
Elapidae, Hydrophianae																
*Acanthophis praelongus*	T					Toad experience+	0.73			Au	Trop	V	M	E	B	Phillips et al. (2010), Webb, Brook, and Shine (2002) (size, maturation)
*Emydocephalus annulatus*	CMR	−	−			Color morph−	0.69			Au	Trop	V	S	E	A	Shine, Brischoux, and Pile (2010), Shine, Shine, and Shine (2003) (size); Masunaga and Ota (2003) (maturation and parity in *E. ijimae*)
*Hoplocephalus bungaroides*	CMR	−	−	−		Pre− vs. postfire−	0.74	0.68		Au	Subt	V	M	L	B	Webb and Shine (2008), Webb, Christian, and Fisher (2002) (size, maturation, parity)
*Notechis scutatus*	CMR	−	+	+			0.79	0.57		Au	Subt	V	L	E	A	Bonnet et al. (2011), Shine (1977) (parity)
*Rhinoplocephalus nigrescens*	CMR	−	−	−		Pre− vs. postfire+	0.61			Au	Subt	V	S	I	A	Webb and Shine (2008), Shine (1984) (size), Webb, Christian et al. (2002) (maturation, parity)
Pythonidae																
*Liasis fuscus*	CMR	−				Prey abundance−, pre− vs. postflood+	0.80			Au	Trop	O	L	E	A	Ujvari et al. (2016), Madsen et al. (2006) (size, parity), Madsen and Shine (2000) (maturation)
Viperidae, Crotalinae																
*Agkistrodon piscivorus*	CMR	−	−	−		Season−, transience+	0.79			NA	Subt	V	L	L	B	Koons, Birkhead, Boback, Williams, and Greene (2009), Conant (1975) (size), Ford (2002) (maturation, parity)
*Agkistrodon piscivorus*	CMR	−	−			Area−	0.81			NA	Subt	V	M	L	B	Rose, Simpson, Ott, and Manning (2010), Rose, Simpson, Ott, Manning, and Martin (2010) (size), Ford (2002) (maturation, parity)
*Bothrops insularus*	CMR		+			Season+	0.49			SA	Trop	V	M	E	B	Guimaraes, Munguia‐Steyer, Doherty, Martins, & Sawaya (2014), Marques, Kasperoviczus, and Almeida‐Santos (2013) (size, parity), Hartmann, Marques, and Almeida‐Santos (2004) (maturation in *B. neuwiedi*)
*Crotalus adamanteus*	CMR/T		−	−		Season+, condition+	0.82			NA	Subt	V	L	L	B	Waldron, Welch, Bennett, Kalinowsky, and Mousseau (2013)
*Crotalus horridus*	CMR	−	−	+		Morph+, marking treatment−, cohort−	0.89	0.84	0.65	NA	Temp	V	L	L	B	Brown et al. (2007), Brown (1991) (size), 1993 (size), 2008
*Crotalus horridus*	T	+	+			Season+, weather−, time since marking+, female reproductive state−, condition−, prey abundance+	0.88			NA	Temp	V	L	L	B	Olson, MacGowan, Hamilton, Currylow, and Williams (2015), Brown (1991) (size), 1993 (size), Brown et al. (2007) (maturation, parity), Brown (2008) (maturation, parity)
*Crotalus oreganus*	CMR	+	−	+			0.82	0.76	0.66	NA	Temp	V	M	E	B	Diller and Wallace (2002)
*Crotalus pricei*	CMR	−					0.71			NA	Subt	V	M	I	B	Prival and Schroff (2012)
*Sistrurus catenatus*	CMR	−	+	+			0.69	0.66	0.38	NA	Temp	V	M	I	B	Hileman (2016), Hileman, King, and Faust (2018)
*Sistrurus catenatus*	CMR	−	−			Transience+	0.74			NA	Temp	V	M	I	B	Jones et al. (2017), Hileman (2016) (size, maturation, parity)
*Sistrurus catenatus*	CMR	−	−				0.94	0.85	0.65	NA	Temp	V	M	I	B	Baker (2016)
*Sistrurus catenatus*	CMR	−					0.79			NA	Temp	V	M	I	B	Johnson (2013), Johnson, Gibbs, Shoemaker, and Cohen (2016), Hileman (2016) (maturation)
*Sistrurus tergeminis*	T				+		0.83			NA	Temp	V	M	I	B	Jones et al. (2012), Seigel (1986) (size, maturation, parity)
*Trimeresurus albolabris*	T					Translocated vs. resident−	0.32			As	Trop	V	M	I	B	Devan‐Song (2014), Nishimura and Kamura (1994) (maturation in *T. flavoviridis*)
Viperidae, Viperinae																
*Bitis schneideri*	CMR			+			0.48	0.42		Af	Trop	V	S	E	A	Maritz (2011), Maritz and Alexander (2012)
*Vipera aspis*	CMR	−	−	+	+	Weather+	0.83	0.68	0.49	E	Temp	V	M	I	B	Flatt, Dummermuth, and Anholt (1997), Altwegg, Dummermuth, Anholt, and Flatt (2005), Naulleau and Bonnet (1995) (size)
*Vipera berus*	CMR	+	−		+	Prey availability+	0.73			E	Temp	V	M	I	B	Forsman and Lindell (1997), Forsman (1995) (size, maturation, parity)
*Vipera ursinii*	CMR		−	−		Habitat−, cohort+, maternal traits−, birth mass+, birth condition+		0.48	0.48	E	Temp	V				Baron, Le Galliard, Tully, and Ferriere (2010)
*Vipera ursinii*	CMR	−	−	−	+	Fire+	0.64			E	Temp	V	S	L	B	Lyet et al. (2009), Baron, Le Galliard, Ferriere, and Tully (2013) (size, maturation, parity)
*Vipera ursinii*	CMR			−		Reproductive state−, postpartum condition−	0.69			E	Temp	V	S	L	B	Baron et al. (2013)

Note

Grouping variables and covariates included in candidate models are indicated by + (top‐ranking models) or − (lower‐ranking models). Estimates of adult, juvenile, and neonate survival represent means across sexes, sites, and years. Studies are classified by continent (NA: North America; E: Europe; SA: South America; Af: Africa; As: Asia; and Au: Australia), climate zone (Temp: temperate; Subt: Subtropical; and Trop: Tropical), reproductive mode (O: oviparous; and V: Viviparous), size (S: small; M: medium; and L: Large), maturation (E: early; I: intermediate; and L: late), and parity (A: annual or more frequent; and B: biennial or less frequent). Sources of size, maturation, and parity are denoted parenthetically if different from sources of survival. Variable definitions are explained more fully in the text; see Supporting Information File S1 for additional information and studies.

**Figure 6:**
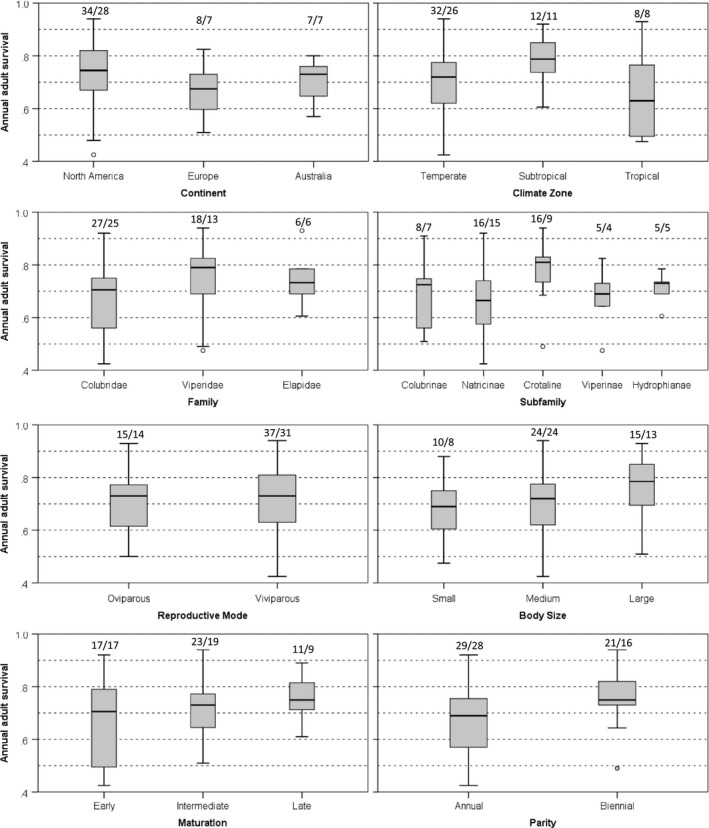
Box plots showing variation in adult snake survival among continents, climate zones, families, subfamilies, modes of reproduction, body size, maturation, and parity. Bars represent medians, boxes represent 50th percentiles, and whiskers represent ranges excluding outliers (points). The number of estimates and the number of species are listed above each box plot

Across studies, estimates of annual adult survival ranged from 0.23 to 0.94 (median = 0.73). Three estimates appeared to be outliers based on their standard *z* scores; *Nerodia harteri* (*ϕ* = 0.23, *z* = −2.81), *Tropidonophis mairii* (*ϕ* = 0.26, *z* = −2.62), and *Trimersurus albolabris* (*ϕ* = 0.32, *z* = −2.26). These three estimates were excluded from further consideration; their exclusion resulted in no change in the median (0.73, range = 0.43–0.94) nor qualitative change in other results. Estimates of annual survival overlapped broadly across continents, climate zones, families, subfamilies, reproductive modes, body size categories, maturation categories, and parity categories (Figure 6). The largest differences in annual survival were seen among climate zones (median = 0.72, 0.78, and 0.63 for temperate, subtropical, and tropical areas, respectively), among families and subfamilies (0.67, 0.79, and 0.73 for Colubridae, Viperidae, and Elapidae; 0.67 and 0.81 for Natricinae and Crotalinae), among size categories (0.69, 0.72, and 0.79 for small, medium, and large snakes), and between parity categories (0.69 vs. 0.75 for annually vs. biennially reproducing snakes). Smaller differences in annual survival were seen among maturation categories (0.71, 0.73, and 0.75 for early, intermediate, and late maturing snakes), among continents (0.73, 0.68, and 0.75 for Australian, European, and North American snakes), and between modes of reproduction (0.73 for both oviparous and viviparous snakes). Annual survival among neonates (median = 0.48, range = 0.16–0.76, *n* = 12) and juveniles (median = 0.57, range = 0.14–0.85, *n* = 18) was generally lower than among adults (Table 2, Supporting Information File S1). No consistent difference in survival between adult males and females was evident (Supporting Information File S1).

Analyses frequently included one or more grouping variables or covariates (year, sex, site, and size/age/stage) in the model selection process (Table 2). These variables appeared among high‐ranking models with varying frequency. For example, survival frequently differed among study sites (nine of 10 studies in which site was included among candidate models) and among size, stage, or age classes (17 of 26 studies). Year and sex appeared among high‐ranking models less frequently (just 14 of 36 and 13 of 38 studies, respectively). Only a single study included size as a continuous covariate. Using a growth function to estimate the size of snakes when not encountered, Hansen, Scherer, White, Dickson, and Fleishman (2015) demonstrated that, for *Thamnophis gigas* (Giant Gartersnake), survival increased as function of SVL from 0.37 among neonates to 0.73 among large adults. Similarly, survival consistently increased with increasing size, stage, or age in studies using these grouping variables.

Survival was frequently associated with environmental factors. In *Thamnophis elegans* (Wandering Gartersnake), adult survival was higher in lakeshore than in meadow populations and covaried with body size, age at first reproduction, reproductive frequency, and food availability (relatively constant at lakeshore sites, highly variable at meadow sites; Bronikowski & Vleck, 2010; Miller, Clark, Arnold, & Bronikowski, 2011). Adult survival was also associated with prey availability in *Liasis fuscus* (Water Python, Madsen, Ujvari, Shine, & Olsson, 2006; Ujvari, Brown, Shine, & Madsen, 2016) and *Vipera berus* (European Adder, Forsman & Lindell, 1997). Changes in adult survival were associated with the arrival of nonnative toxic toads in the *Tropidonophis mairii* (Common Keelback) and *Acanthophis praelongus* (Northern Death Adder) but not *Stegonotus cucullatus* (Slatey‐grey Snake; Brown, Ujvari, Madsen, & Shine, 2013; Phillips, Greenlees, Brown, & Shine, 2010). Other variables associated with variation in survival included degree of habitat fragmentation (*Drymarchon couperi*, Indigo Snake, Breininger et al., 2012), fire (*Vipera ursinii*, Ursini’s Viper; *Rhinoplacephalus nigrescens,* Small‐eyed Snake; Webb & Shine, 2008; Lyet, Cheylan, Prodon, & Besnard, 2009), color morph (*Vipera berus,* European Adder; *Crotalus horridus,* Timber Rattlesnake; Forsman, 1995; Brown, Kery, & Hines, 2007; Brown, 2008), and possibly, disturbance by researchers (*Crotalus oreganus,* Pacific Rattlesnake; Diller & Wallace, 2002).

## 4 DISCUSSION

### 4.1 Population size and realized population growth

Population size of the Lake Erie Watersnake is of interest for two reasons. The first is the sheer density that this snake achieves. Descriptions by French explorers of watersnakes “sunning themselves in heaps, knots, and snarls” (Ballou, 1878) and by mid‐twentieth‐century biologists of catching hundreds in a single day (Conant, 1997) seemed steeped in hyperbole when efforts to quantify population density began in the 1980s (King, 1986). Currently, watersnake density varies from 160 to 1,600 adults per km of shoreline among study sites (mean = 520/km, Table 1), a dramatic increase from early quantitative estimates (King, 1986; King, Queral‐Regil, et al., 2006). Furthermore, capture rates exceeding 100 adults per site per day during annual population censuses are once again a regular occurrence, suggesting that past abundance has been regained at many sites.

Lake Erie Watersnakes occupy a narrow band of shoreline habitat during the active season (90% of on‐shore activity occurs within 21 m of shore, Stanford et al., 2010). Thus, linear density estimates translate into ca. 260 adults per ha (range ≈ 80–800 adults per ha). In contrast, densities of Northern Watersnakes (*N. s. sipedon*) occupying marshes and ponds in southern Michigan and southern Ontario are an order of magnitude lower (26 adults per ha, Feaver, 1977; 25 and 33 adults per ha, Brown & Weatherhead, 1999), as is an introduced population in California (15 adults per ha estimated from Figure 3 in Rose, Miano, & Todd, 2013). Among congeners, *N. fasciata* achieves a density of 45 adults per ha in a South Carolina bay (Willson & Winne, 2016), and *N. taxispilota* achieves a density of 200 juveniles and adults per km of the Savannah River (Mills, 2002). To our knowledge, only two other snake species similar in body size to the Lake Erie Watersnake achieve comparable densities. *Natrix tessellata* has a density >500/ha at a study site in Macedonia (Ajtic et al., 2013), and *Gloydius shedaoensis* has a density of ca. 200/ha at a study site in China (Shine, Sun, Kearney, & Fitzgerald, 2002). Both involve island populations. Like the Lake Erie Watersnake, *N. tessellata* feeds on locally abundant fish, whereas *G. shedaoensis* feeds on temporally abundant migratory birds.

Lake Erie Watersnake population size is also of practical interest; criteria for delisting and for postdelisting monitoring both included island‐specific and total adult population size targets (U.S. Fish and Wildlife Service, 2003, 2011). Summing population estimates across sites within islands and across all 14 study sites (Table 1) demonstrate that these targets have been met. In addition to population size criteria, the Lake Erie Watersnake postdelisting monitoring plan included analyses of realized population growth (U.S. Fish and Wildlife Service, 2011). Specifically, the plan specified that 95% confidence intervals for *λ* that included or exceeded one represented evidence of stable or increasing population size. Taken together, estimates of population size and realized population growth indicate that, during postdelisting monitoring, the species has remained secure without Endangered Species Act protections (U.S. Fish and Wildlife Service, 2011).

### 4.2 Annual survival and process variance in annual survival

Our analyses indicate that annual adult survival of the Lake Erie Watersnake is relatively high (ca. 0.72), is greater among males than females (0.76 vs. 0.68), and varies among sites (by 0.18 in males and 0.27 in females). Estimates provided by the CJS model are of “apparent survival,” the product of survival and emigration probability. Thus, some of the variation in survival that we see among sites could arise from variable emigration rates. However, high site fidelity of adult Lake Erie Watersnakes, as indicated by our recapture data (99.4% of recaptures occurred within study sites) and radio telemetry (Stanford et al., 2010), suggests that emigration is infrequent and likely has only minor effects on survival estimates.

In contrast, our top‐ranked model suggests that variation in survival among years is of lesser significance. Temporal process variance averaged just 0.0011, less than 4% of total variance. This has implications for population projections and models of extinction risk that include stochasticity (Akçakaya & Root, 2002; Lacy & Pollak, 2014). In the absence of estimates like ours, total variance or arbitrary values (e.g., 10%) are sometimes used in place of process variance, potentially resulting in overly pessimistic assessments of extinction risk (Jones, King, & Sutton, 2017). Long‐term data (10 or more years) are needed to calculate temporal process variance (Gould & Nichols, 1998; Burnham & Anderson, 2002; Anderson, 2008) and so estimates are scarce. However, those estimates for snakes that do exist are uniformly low (Table 3) suggesting that stochasticity in annual survival may be of minor significance to snake extinction risk. In cases where process variance cannot be estimated directly, values in Table 2 might serve as a proxy.

**Table 3:** Estimates of temporal process variance in snake annual survival

Species	Temporal process variance in annual survival			Citation
	Adults	Juveniles	Neonates	
*Contia tenuis*	0.073			Govindarajulu et al. (2011)
*Nerodia harteri*	0.007–0.01	0.006		Whiting et al. (2008)
*Nerodia sipedon*	<0.0001–0.0080	0.032		This study (adults), Stanford (2012) (juveniles)
*Sistrurus catenatus*	0.006			Jones et al. (2017)
*Thamnophis elegans*	<0.0001–0.003	<0.0001–0.002	0.0001–0.003	Bronikowski & Arnold (1999), Miller et al. (2011)
*Vipera ursinii ursinii*			0.073	Baron et al. (2010)
*Natrix tesselata*	0.0053			Calculated from Luiselli et al. (2011) (following Gould & Nichols, 1998)

We found that adult survival was positively correlated with body size (mean SVL of the largest 10% of animals) across sites in both males and females. Interpreting this pattern is complicated by extensive individual variation in the asymptotic size of Lake Erie Watersnakes (King et al., 2016) such that larger individuals are not necessarily older. Consequently, it is unclear whether high survival leads to increased body size, large body size leads to increased survival, or some other mechanism underlies variation in both size and survival. Given that our 14 study sites are separated by less than 20 km, variation in climatic conditions is likely to be small and certainly far less than that seen in range‐wide demonstrations of variation in survival (Jones et al., 2012) or body size (Hileman, King, et al., 2017; Hileman, Powell, et al., 2017). Study sites differ in the extent of human activity and may differ in predator abundance, but how these variables affect mortality awaits further investigation. Prey availability or foraging success may also differ among sites, and this could contribute to variation in both survival rates (e.g., through an effect on body condition) and size (e.g., through an effect on growth). Cases in which survival varies as a consequence of prey availability are well documented in our review of snake survival, suggesting that further investigation of this linkage in Lake Erie Watersnakes would be worthwhile.

### 4.3 Review of snake survival

Estimates of snake adult survival overlap broadly across continents, climate zones, families, subfamilies, reproductive modes, body size categories, maturation categories, and parity categories. There is some evidence for higher survival in subtropical (median = 0.78) vs. tropical areas (0.63), among Viperidae (0.79) vs. Colubridae (0.67), especially Crotalinae (0.81) vs. Natricinae (0.67), in large‐ (0.79) vs. small‐bodied snakes (0.69), in biennially (0.75) vs. annually (0.69) reproducing snakes, and in later (0.75) vs. earlier (0.71) maturing snakes. Notably, differences in survival in relation to size, parity, and maturation are in the directions predicted by life history theory (Roff, 1992; Stearns, 1992) but are of small magnitude with much variation around median values. In contrast to Parker and Plummer (1987, tables 9‐5), we found that annual survival differed little among early maturing temperate colubrids (median adult survival = 0.67, range = 0.43–0.79, *n* = 7), late maturing temperate colubrids (median = 0.66, range = 0.51–0.75, *n* = 13), and late maturing temperate viperids (median = 0.70, range = 0.64–0.94, *n* = 11; Table 2).

Covariation in demographic traits is well documented in mammals, birds, lizards, and fishes (e.g., Gaillard et al., 1989, 2005; Mesquita, Faria, Colli, Vitt, & Pianka, 2016; Mesquita, Costa, et al., 2016; Promislow & Harvey, 1990; Winemiller & Rose, 1992), but whether this covariation conforms to a single (slow‐fast) axis or two (opportunistic–periodic–equilibrium) axes (Dunham & Miles, 1985; Winemiller, 2004; Bielby et al., 2007) and the ways in which demography covaries with physiology and behavior remain areas of active research (Pianka et al., 2017; Réale et al., 2010; Ricklefs & Wikelski, 2002; Winemiller et al., 2015). At the same time, species or larger taxonomic groups that represent exceptions to general patterns provide interesting insights regarding demographic variability. For example, crocodilians and turtles exhibit a suite of demographic traits that are unusual among vertebrates; delayed maturity and high adult survival coupled with high fecundity (BriggsGonzalez et al., 2017). The variation in annual survival revealed in our case study, and review suggests that demography may be unusually plastic in snakes compared to more frequently studied taxa. Our review reveals a strong bias favoring North American and temperate/subtropical species with few representatives from centers of snake diversity in the tropics (compare Figure 6 with figure 1d in Roll et al., 2017), thus much remains to be learned regarding snake demography. We suspect that, as additional studies accumulate, future analyses, which might also include juvenile survival, offspring size, offspring number, growth parameters, and generation time, will clarify ecological and evolutionary determinants of snake life history. Additional studies are also likely to provide further information on the magnitude of process variance in annual survival, possibly confirming our suggestion, based on existing examples, that such variance is generally low. This has practical implications for making accurate projections of future population trajectories (e.g., for species of conservation concern) and may offer insight into the stability of snake population dynamics more generally.

## AUTHOR CONTRIBUTIONS

All authors conceived the ideas, designed methodology, analyzed the data, wrote the manuscript, contributed to revisions, and gave final approval for publication.

## DATA ACCESSIBILITY

Data available from the Dryad Digital Repository: https://doi.org/10.5061/dryad.kh022n1
