# Supplementary material for: Sunning themselves in heaps, knots, and snarls: The extraordinary abundance and demography of island watersnakes
Source: Ecol Evol. 2018 Jul 4;8(15):7500–21. doi: 10.1002/ece3.4191 (PMC6106160; doi:10.1002/ece3.4191)
Supplement: Supplementary file 1 [file ECE3-8-7500-s001.docx]

**Supporting Information for**

**Variation in Snake Demography:**

**A Case Study and Synthesis**

**Richard B. King ^* a,b^, Kristin M. Stanford ^c^, and Peter C. Jones ^a^**

^a^ Department of Biological Sciences, Northern Illinois University, DeKalb, IL 60115

^b^ Institute for the Study of the Environment, Sustainability and Energy, Northern Illinois University, DeKalb, IL 60115

^c^ F. T. Stone Laboratory, Ohio State University, Put-in-Bay, OH 43456

^*^ Corresponding author: rbking@niu.edu

**Data S1 Error Checking**

Correctly identifying marked animals was a primary goal of snake processing. However, in a study of this magnitude, involving thousands of capture records collected over many years by large numbers of participants, some errors are inevitable. The use of PIT tags to permanently and uniquely identify individual animals goes far in minimizing errors but does not eliminate them as detailed below:

Tag loss: On six occasions, we recaptured a snake a few days after it had been tagged (as evident from the presence of a paint mark) but failed to detect a tag. Presumably, these snakes lost their tag before the injection site had healed.

Tag failure: On three occasions we recaptured snakes in which we detected the physical presence of a tag under the skin from which no tag code could be recovered. We attribute this to tag failure.

Detection failure: We know of five animals that were retagged after an existing tag went undetected. We attribute this to incompletely scanning an animal prior to tagging

Scanner failure: On a few occasions, PIT tag scanner memory registers became corrupted, resulting in the same tag code being assigned to multiple snakes.

Observer error: Tag codes and capture status (new capture, recapture) were sometimes recorded incorrectly or illegibly on handwritten field data sheets.

Comparison of written records with registers of tag codes provided by the supplier and information obtained from subsequent recaptures allowed us to resolve most errors. The net result was that tag codes were unknown for 70 individually marked animals (0.5% of all marked snakes) and prior captures were unresolved for 85 recaptures (0.9% of all recaptures). For analysis, animals whose prior capture was unresolved were treated as new captures. Errors in recording size (SVL, mass) or sex of animals were apparent when values fell out-of-range for a given variable (e.g., from plots of length-mass relationships) or when discrepancies were noted for animals captured multiple times (e.g., sex recorded inconsistently). When these errors could not be resolved by examining field data sheets, information from recaptures, or knowledge of animal biology (e.g., males rarely exceed 800 mm SVL and 300 g), they were treated as missing values. Cases for which sex was unknown (52 captures of 27 animals) were excluded from comparisons of males and females. Cases for which age (sub-adult, adult) was unknown (17 captures of 15 animals of unknown sex falling between 430 and 590 mm SVL) were excluded from analyses of adults.

**Data S2 Permanence of Emigration**

We identified 105 cases involving 100 individual Lake Erie Watersnakes that moved between sites (Table S5). Five cases involved recovery of the carcass of a dead snake that may have drifted from one site to another. Eighteen other cases appear to represent ‘unplanned translocations’ that occurred when snakes were released at the incorrect site following processing. Unplanned translocation could be inferred when (1) multiple snakes captured on the same date and same site were later recaptured at a different study site, (2) those sites had been visited on the same day or on successive days, and (3) snakes from those sites were transported to the F. T. Stone Laboratory or South Bay for processing. Six such incidents, involving two or three animals each, were identified. Unplanned translocation was also inferred in one case of an escaped animal used in feeding trials (Jones et al. 2008) and later recaptured outside the building where feeding trials occurred. Of the remaining 82 cases, 57 involved moves among our 14 intensive study sites, 14 involved moves from intensive to less-intensive study sites, 9 involved moves from less-intensive to intensive study sites and 2 involved moves among less-intensive study sites. Two animals were captured at three different sites and three animals returned to their prior site of capture after having been recaptured at a different site (one via an unplanned translocation). However, in only one case did an animal move back and forth between two intensive study sites. In contrast to the infrequent occurrence of moves between study sites, there were 9,358 cases of recaptures that occurred at the same site as prior capture (excluding 125 animals that were dead when recaptured and 85 animals whose prior capture was unresolved). Thus, only 0.6% of recaptures involved moves among intensive study sites. The high site fidelity of adult Lake Erie Watersnakes suggested by these recapture data is consistent with movement patterns observed using radio telemetry (Stanford et al. 2010).

The shortest over-water distance separating study sites between which adult Lake Erie Watersnakes moved ranged from 0 (between adjacent sites) to 35 km, although in only one case did this distance exceed 10.3 km (Fig. S1). It is possible that some moves between sites represent human transport; recreational boating and angling are popular in the region and Lake Erie Watersnakes sometimes board boats docked at area marinas (pers. obs.). For example, a 35 km move from East Point on Middle Bass Island to Point Pelee on the Ontario mainland and a 10.3 km move from Kelleys Island to Middle Island, Ontario both originated from sites with small private marinas. However, 9-10 km moves by three snakes from the East Shore of South Bass Island, a site lacking marinas or docks, to Kelleys Island suggest that long-distance moves sometimes occur naturally.

Snakes involved in unplanned translocations took up residence at their release site as indicated by subsequent recaptures which occurred with equal frequency to those of animals that made unassisted moves between intensive study sites (mean number of recaptures = 1.9 vs 1.5, range = 1-8 vs. 1-5, n = 17 vs. 57; Kolmogorov-Smirnov Test P = 0.991).

**Fig. S1.** Shortest over-water distance separating study sites between which adult Lake Erie Watersnakes moved. Moves between intensive study sites are shaded black (n = 57); moves between an intensive study site and a less-intensive study site or between two less intensive study sites are unshaded (n = 25). Unplanned translocations are excluded. **
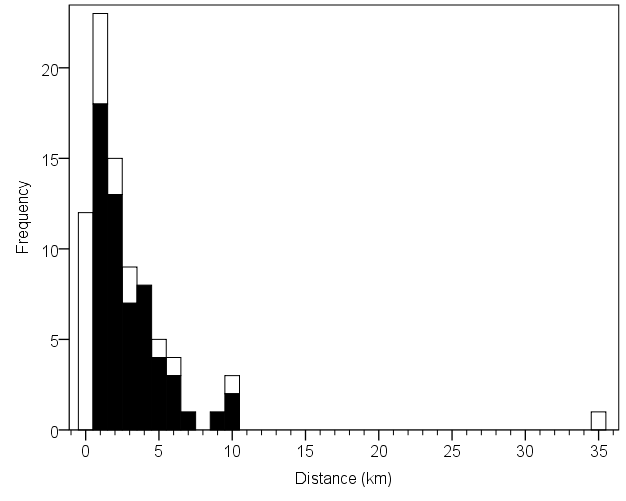
**

**Fig. S2.** Estimated population size of adult male (unfilled circles) and female (filled circles) Lake Erie Watersnakes and associated 95% confidence intervals. Population size is plotted on a log scale to accommodate broad confidence intervals. Study sites, represented by separate panels, include A. Kelleys Island (KI) Long Point, B. KI SE Shore, C. KI South Shore, D. KI Minshall, E. KI State Park, F. South Bass Island (SBI) East Point, G. SBI East Shore, H. SBI State Park, I. Middle Bass Island (MBI) East Point, J. MBI State Park, K. MBI West End, L. North Bass Island (NBI) NE,E,SE Shore, M. NBI South Shore, and N. Gibraltar Island.

**Fig. S3.** Estimated annual recapture probabilities, p, of adult male (unfilled circles) and female (filled circles) Lake Erie Watersnakes and associated 95% confidence intervals. Study sites, represented by separate panels, include A. Kelleys Island (KI) Long Point, B. KI SE Shore, C. KI South Shore, D. KI Minshall, E. KI State Park, F. South Bass Island (SBI) East Point, G. SBI East Shore, H. SBI State Park, I. Middle Bass Island (MBI) East Point, J. MBI State Park, K. MBI West End, L. North Bass Island (NBI) NE,E,SE Shore, M. NBI South Shore, and N. Gibraltar Island.

**Table S1.** Candidate Cormack-Jolly-Seber models for annual survival (φ) and recapture probability (p) of adult Lake Erie Watersnakes. The number of parameters was calculated to account for variation in first and last year of sampling among sites and confounding due to the inclusion of time (Burnham and Anderson 2002).

| **Model** | **Number of Parameters** | **AICc** | **ΔAICc** | **Weight** | **Deviance** |
| --- | --- | --- | --- | --- | --- |
| Φ(sex*site)p(site*time+sex) | 221 | 36646.10 | 0.00 | 0.76 | 7536.42 |
| Φ(sex*site+time)p(site*time+sex) | 234 | 36648.42 | 2.32 | 0.24 | 7512.07 |
| Φ (sex*site)p(site*time) | 220 | 36662.90 | 16.80 | 0.00 | 7555.26 |
| Φ (sex*site+time)p(site*time) | 233 | 36666.56 | 20.46 | 0.00 | 7532.26 |
| Φ (sex+site)p(sex*site*time) | 399 | 36700.50 | 54.40 | 0.00 | 7222.26 |
| Φ (sex*site)p(sex*site*time) | 412 | 36705.92 | 59.81 | 0.00 | 7200.46 |
| Φ (sex+site+time)p(sex*site*time) | 412 | 36708.38 | 62.27 | 0.00 | 7202.92 |
| Φ (sex*site+time)p(sex*site*time) | 425 | 36714.05 | 67.95 | 0.00 | 7181.35 |
| Φ (site)p(sex*site*time) | 398 | 36717.31 | 71.21 | 0.00 | 7241.16 |
| Φ (site+time)p(sex*site*time) | 411 | 36724.74 | 78.64 | 0.00 | 7221.38 |
| Φ (sex+site)p(site*time+sex) | 208 | 36768.49 | 122.39 | 0.00 | 7685.43 |
| Φ (sex+site+time)p(site*time+sex) | 221 | 36771.23 | 125.13 | 0.00 | 7661.54 |
| Φ (sex+site)p(site*time) | 207 | 36774.89 | 128.79 | 0.00 | 7693.89 |
| Φ (sex+site+time)p(site*time) | 220 | 36779.60 | 133.50 | 0.00 | 7671.97 |
| Φ (sex)p(sex*site*time) | 386 | 36793.26 | 147.16 | 0.00 | 7342.18 |
| Φ (sex+time)p(sex*site*time) | 399 | 36799.42 | 153.32 | 0.00 | 7321.18 |
| Φ (site)p(site*time) | 206 | 36801.79 | 155.69 | 0.00 | 7722.83 |
| Φ (site)p(site*time+sex) | 207 | 36802.85 | 156.75 | 0.00 | 7721.84 |
| Φ (sex*time)p(sex*site*time) | 412 | 36805.60 | 159.50 | 0.00 | 7300.15 |
| Φ (site+time)p(site*time) | 219 | 36807.48 | 161.38 | 0.00 | 7701.90 |
| Φ (site+time)p(site*time+sex) | 220 | 36808.33 | 162.23 | 0.00 | 7700.69 |
| Φ (time)p(sex*site*time) | 398 | 36842.91 | 196.81 | 0.00 | 7366.75 |
| Φ (sex)p(site*time+sex) | 195 | 36847.28 | 201.18 | 0.00 | 7790.82 |
| Φ (sex+time)p(site*time+sex) | 208 | 36847.78 | 201.68 | 0.00 | 7764.72 |
| Φ (sex*time)p(site*time+sex) | 221 | 36859.53 | 213.43 | 0.00 | 7749.85 |
| Φ (site*time)p(sex*site*time) | 562 | 36861.67 | 215.57 | 0.00 | 7039.36 |
| Φ (sex)p(site*time) | 194 | 36865.13 | 219.03 | 0.00 | 7810.71 |
| Φ (sex+time)p(site*time) | 207 | 36867.72 | 221.62 | 0.00 | 7786.71 |
| Φ (sex*time)p(site*time) | 221 | 36880.47 | 234.36 | 0.00 | 7770.78 |
| Φ (time)p(site*time) | 206 | 36913.26 | 267.16 | 0.00 | 7834.30 |
| Φ (time)p(site*time+sex) | 207 | 36915.18 | 269.08 | 0.00 | 7834.17 |
| Φ (site*time)p(site*time+sex) | 371 | 36938.95 | 292.85 | 0.00 | 7519.17 |
| Φ (site*time)p(site*time) | 370 | 36939.57 | 293.47 | 0.00 | 7521.87 |
| Φ (sex*site*time)p(site*time+sex) | 563 | 36974.30 | 328.20 | 0.00 | 7149.85 |
| Φ (sex*site*time)p(site*time) | 562 | 36986.27 | 340.17 | 0.00 | 7163.95 |
| Φ (sex*site*time)p(sex*site*time) | 740 | 37070.05 | 423.95 | 0.00 | 6864.61 |

**Table S2.** Candidate Pradel models for realized population growth (λ) of adult Lake Erie Watersnakes. For all models, survival and recapture probabilities were modeled as φ(sex*site)p(site*time+sex).

| **Model** | **Number of Parameters** | **AICc** | **ΔAICc** | **Weight** | **Deviance** |
| --- | --- | --- | --- | --- | --- |
| λ(site*sex+time) | 261 | 103514.98 | 0.00 | 1.00 | 8212.90 |
| λ(site*sex) | 249 | 103525.75 | 10.77 | 0.00 | 8248.33 |
| λ(site+sex*time) | 262 | 103526.34 | 11.37 | 0.00 | 8222.22 |
| λ(site*sex*time) | 603 | 103540.27 | 25.29 | 0.00 | 7522.54 |
| λ(site+time) | 247 | 103573.05 | 58.07 | 0.00 | 8299.73 |
| λ(site+sex+time) | 248 | 103574.37 | 59.39 | 0.00 | 8298.99 |
| λ(site) | 235 | 103582.55 | 67.57 | 0.00 | 8333.84 |
| λ(site+sex) | 236 | 103583.99 | 69.01 | 0.00 | 8333.23 |
| λ(site*time+sex) | 412 | 103667.00 | 152.02 | 0.00 | 8052.14 |
| λ(site*time) | 411 | 103667.12 | 152.15 | 0.00 | 8054.35 |

**Table S3.** Numbers of adult Lake Erie Watersnakes captured, checked for marks, marked, and released at 14 intensive study sites, 1996-2015 (numbers in parentheses denote additional snakes that were dead or released unmarked). ‘Length’ refers to the extent of shoreline (in km) included within a given study site.

|  | Kelleys Island | | | | | South Bass Island | | | Middle Bass Island | | | North Bass Island | | Gibraltar  Island |
| --- | --- | --- | --- | --- | --- | --- | --- | --- | --- | --- | --- | --- | --- | --- |
|  | Long  Point | Southeast  Shore | South  Shore | Minshall | State  Park | East  Point | East  Shore | State  Park | East  Point | State  Park | West  End | NE,E,SE  Shore | South  Shore |  |
| Length (km) | 2.8 | 1.0 | 0.7 | 1.3 | 0.7 | 1.3 | 1.0 | 0.7 | 1.0 | 1.0 | 1.1 | 2.4 | 2.0 | 0.9 |
| 1996 |  |  | 15 | 19 (7) | 8 (5) | 3 |  | 21 (1) | 12 | 9 | 17 (6) | 1 | 15 (2) | 5 |
| 1997 |  |  | 10 (2) | 23 (2) | 8 (1) | 5 |  | 24 | 40 | 5 | 14 (3) | 1 | 7 | 7 |
| 1998 |  |  | 3 | 9 (1) | 22 |  |  | 53 | 5 | 11 | 13 |  | 11 |  |
| 1999 |  |  |  |  |  |  |  |  |  |  |  |  |  |  |
| 2000 | 16 |  | 8 | 15 | 11 |  |  |  |  |  |  |  |  |  |
| 2001 | 30 | 182 | 123 | 36 | 33 | 6 | 4 | 26 | 1 | 5 (1) | 12 |  | 14 | 2 |
| 2002 | 32 | 162 | 107 (1) | 3 | 41 |  | 124 | 93 |  | 31 | 125 |  | 49 |  |
| 2003 | 1 | 118 | 114 | 5 | 74 (1) |  | 102 | 218 | 4 | 71 | 103 |  | 58 | 32 |
| 2004 | 9 | 160 (3) | 73 | 50 (1) | 63 (1) |  | 61 (4) | 169 (4) | 39 | 78 (1) | 140 (1) | 35 | 74 (1) | 61 (1) |
| 2005 | 3 | 128 (1) | 104 | 26 | 33 | 50 | 115 | 272 (4) | 96 (1) | 41 (2) | 96 (5) | 44 | 67 (4) | 63 |
| 2006 | 18 | 138 (1) | 136 (1) | 35 (1) | 104 | 40 | 155 | 261 (7) | 86 (2) | 65 | 85 (8) | 41 | 36 (2) | 33 |
| 2007 | 53 (2) | 168 (2) | 137 (1) | 24 (1) | 59 (3) | 57 | 185 | 322 (4) | 139 (3) | 160 (4) | 144 | 105 | 157 (3) | 81 |
| 2008 | 84 (1) | 124 (1) | 136 (1) | 17 | 56 (1) | 51 | 158 | 150 (2) | 99 (9) | 288 (10) | 102 (1) | 38 | 93 | 23 (15) |
| 2009 | 77 (1) | 135 (1) | 149 | 28 | 45 | 81 | 225 (1) | 207 (2) | 108 (1) | 156 (3) | 66 (6) | 77 (3) | 137 | 78 (1) |
| 2010 | 49 | 199 | 194 | 9 | 50 (1) | 154 (2) | 173 | 157 (6) | 110 (1) | 82 (3) | 57 | 104 | 138 (1) | 59 (3) |
| 2011 | 47 (1) | 160 (2) | 248 (3) | 31 (3) | 56 (1) | 145 (4) | 98 | 142 (4) | 121 (4) | 104 (1) | 40 (5) | 46 (4) | 114 (1) | 56 (1) |
| 2012 | 60 | 144 | 222 | - | 65 | 187(1) | 54 | 143 (2) | 100 | 130 (1) | 25 | 88 | 209 | 73 |
| 2013 | 74 | 154 (2) | 208 | - | 88 | 234 (3) | 126 (2) | 184 (2) | 82 | 107 (2) | - | 209 | 412 (4) | 68 (2) |
| 2014 | 56 | 124 (1) | 223 | - | 107 (1) | 129 (1) | 92 (5) | 158 (7) | 61 (2) | 113 (1) | - | 56 (1) | 211 (2) | 91 (2) |
| 2015 | 52 | 241 | 237 (1) | - | 98 (1) | 106 | 62 (1) | 94 (1) | 66 | 67 | - | 84 | 122 | 64 (2) |
| Total Captures | 661 (5) | 2337 (14) | 2447 (10) | 330 (16) | 1020 (16) | 1234 (11) | 1734 (13) | 2694 (46) | 1111 (23) | 1498 (29) | 1039 (35) | 927 (8) | 1924 (20) | 796 (27) |
| Unique Individuals | 483 | 1530 | 1854 | 284 | 631 | 809 | 1191 | 1425 | 879 | 1075 | 862 | 717 | 1398 | 520 |

**Table S4.** Characteristics of capture histories of adult Lake Erie Watersnakes. Captures per Individual Snake refers to the number of different years a given individual was captures (ignoring multiple captures within years), Gap Between Successive Captures refers to the number of years in which a snake went uncaptured between successive captures. A snake caught in two successive years has a gap of 0. Time Span Encompassed by First and Last Capture refers to the number of years over which a snake was known to be present. A snake captured in only one year has a time span of one.

| Captures per Individual Snake | | Gap Between Successive Captures | | Time Span Encompassed by First and Last Capture | |
| --- | --- | --- | --- | --- | --- |
| Years | Frequency | Years | Frequency | Years | Frequency |
| 1 | 9571 | 0 | 3295 | 1 | 9571 |
| 2 | 2653 | 1 | 1470 | 2 | 1172 |
| 3 | 877 | 2 | 731 | 3 | 884 |
| 4 | 319 | 3 | 366 | 4 | 640 |
| 5 | 122 | 4 | 214 | 5 | 508 |
| 6 | 55 | 5 | 100 | 6 | 340 |
| 7 | 18 | 6 | 46 | 7 | 202 |
| 8 | 3 | 7 | 17 | 8 | 138 |
| 9 | 1 | 8 | 15 | 9 | 92 |
|  |  | 9 | 6 | 10 | 41 |
|  |  | 10 | 0 | 11 | 15 |
|  |  | 11 | 1 | 12 | 7 |
|  |  | 12 | 1 | 13 | 4 |
|  |  |  |  | 14 | 4 |
|  |  |  |  | 15 | 1 |

**Table S5.** Numbers of recaptures of adult Lake Erie Watersnakes from 1996-2015 occurring at the site of prior capture (diagonal shaded entries) vs. a different site (off diagonal entries). Intensive study sites are named; less-intensive sites are pooled as “Other Sites.” Rectangles identify study sites on the same island. Abbreviations: KI – Kelleys Island, SBI – South Bass Island, MBI – Middle Bass Island, NBI – North Bass Island. Animals that were dead when recaptured, that were unplanned translocations, or whose prior capture was unresolved were excluded. Total includes off-diagonal entries only.

|  |  | Current Site | | | | | | | | | | | | | | | |
| --- | --- | --- | --- | --- | --- | --- | --- | --- | --- | --- | --- | --- | --- | --- | --- | --- | --- |
|  |  | KI Long Point | KI Southeast Shore | KI South Shore | KI Minshall | KI State Park | SBI East Point | SBI East Shore | SBI State Park | MBI East Point | MBI State Park | MBI West End | NBI N,NE,E Shore | NBI South Shore | Gibraltar Island | Other Sites | Total |
| Prior Site | KI Long Point | 190 |  |  |  |  |  |  |  |  |  |  |  |  |  |  | 0 |
|  | KI SE Shore |  | 1007 |  |  |  |  |  |  |  |  |  |  |  |  |  | 0 |
|  | KI South Shore | 1 | 1 | 701 |  | 3 |  |  |  |  |  |  |  |  |  |  | 5 |
|  | KI Minshall |  |  |  | 71 | 1 |  |  |  |  |  |  |  |  |  |  | 1 |
|  | KI State Park | 3 |  |  | 1 | 589 |  |  |  |  |  |  |  |  |  | 1 | 5 |
|  | SBI East Point |  |  |  |  | 1 | 690 |  |  |  | 3 |  |  |  |  | 1 | 5 |
|  | SBI East Shore |  |  | 1 |  | 1 |  | 616 | 1 |  |  |  |  |  | 1 | 1 | 5 |
|  | SBI State Park |  |  |  |  |  | 1 |  | 2492 |  | 3 | 2 |  | 1 | 4 | 3 | 14 |
|  | MBI East Point |  |  |  |  |  | 1 |  |  | 268 | 1 |  |  | 1 | 1 | 1 | 5 |
|  | MBI State Park |  |  |  |  |  |  |  |  |  | 798 |  |  |  | 2 | 4 | 6 |
|  | MBI West End |  |  |  |  |  |  |  | 1 |  | 4 | 250 |  | 3 | 1 | 1 | 10 |
|  | NBI N,NE,E Shore |  |  |  |  |  |  |  |  |  |  |  | 299 | 3 | 1 |  | 4 |
|  | NBI South Shore |  |  |  |  |  |  |  |  |  |  |  | 9 | 910 |  |  | 9 |
|  | Gibraltar Island |  |  |  |  |  |  |  |  |  |  |  |  |  | 477 | 2 | 2 |
|  | Other Sites | 1 |  |  |  | 2 | 1 |  |  |  |  | 1 | 1 |  | 3 | 2 | 11 |
|  | Total | 5 | 1 | 1 | 1 | 8 | 3 | 0 | 2 | 0 | 11 | 3 | 10 | 8 | 13 | 16 | 82 |

**Table S6.** Estimated population sizes (N) and associated 95% confidence intervals (CI) for adult female and male Lake Erie Watersnakes.

|  |  | Kelleys Island | | | | | | | | | | South Bass Island | | | | | | Middle Bass Island | | | | | | North Bass Island | | | | Gibraltar | |
| --- | --- | --- | --- | --- | --- | --- | --- | --- | --- | --- | --- | --- | --- | --- | --- | --- | --- | --- | --- | --- | --- | --- | --- | --- | --- | --- | --- | --- | --- |
|  |  | Long Point | | Southeast Shore | | South Shore | | Minshall | | State Park | | East Point | | East Shore | | State Park | | East Point | | State Park | | West End | | NE,E,SE Shore | | South Shore | |  |  |
| Sex | Year | N | CI | N | CI | N | CI | N | CI | N | CI | N | CI | N | CI | N | CI | N | CI | N | CI | N | CI | N | CI | N | CI | N | CI |
| Female | 2001 | 150 | 50-890 |  |  |  |  |  |  |  |  |  |  |  |  |  |  |  |  |  |  |  |  |  |  |  |  |  |  |
| Female | 2002 | 140 | 70-500 | 160 | 120-250 | 360 | 160-1440 |  |  | 30 | 10-110 |  |  |  |  | 100 | 80-190 |  |  |  |  |  |  |  |  | 120 | 50-600 |  |  |
| Female | 2003 |  |  | 160 | 110-290 | 210 | 90-870 |  |  | 70 | 50-130 |  |  | 180 | 90-540 | 170 | 140-240 |  |  | 40 | 30-110 | 990 | 300-6150 |  |  | 80 | 60-200 |  |  |
| Female | 2004 |  |  | 330 | 230-590 | 530 | 130-4680 |  |  | 50 | 40-90 |  |  | 350 | 140-1380 | 130 | 120-170 |  |  | 210 | 100-740 | 270 | 150-640 |  |  | 170 | 100-410 | 50 | 30-110 |
| Female | 2005 |  |  | 390 | 260-770 | 140 | 50-670 | 70 | 10-1070 | 70 | 40-150 |  |  | 100 | 60-230 | 190 | 170-230 | 50 | 30-210 | 90 | 50-260 | 260 | 140-680 | 150 | 50-900 | 90 | 60-160 | 80 | 50-160 |
| Female | 2006 |  |  | 300 | 210-550 | 180 | 70-780 | 70 | 20-1220 | 90 | 60-160 | 80 | 20-820 | 340 | 210-760 | 180 | 160-220 | 230 | 90-1110 | 150 | 90-390 | 790 | 160-9320 | 340 | 130-1520 | 240 | 110-750 | 50 | 30-70 |
| Female | 2007 | 70 | 50-150 | 260 | 190-410 | 260 | 100-1250 | 50 | 10-1250 | 70 | 40-160 | 40 | 30-110 | 440 | 270-930 | 160 | 150-180 | 410 | 180-1510 | 330 | 220-670 | 340 | 170-1090 | 140 | 100-280 | 200 | 140-360 | 80 | 60-130 |
| Female | 2008 | 190 | 120-390 | 300 | 200-570 | 290 | 130-1130 | 10 | 0-90 | 70 | 40-170 | 620 | 180-4010 | 430 | 270-930 | 180 | 150-230 | 450 | 190-1720 | 250 | 210-330 | 1640 | 310-21050 | 110 | 70-250 | 150 | 120-230 | 80 | 40-250 |
| Female | 2009 | 120 | 80-220 | 260 | 180-470 | 340 | 130-1540 | 30 | 10-140 | 50 | 30-120 | 160 | 90-380 | 710 | 420-1610 | 160 | 140-200 | 390 | 170-1470 | 200 | 160-290 | 140 | 60-630 | 200 | 120-470 | 240 | 180-390 | 80 | 60-120 |
| Female | 2010 | 110 | 80-210 | 220 | 170-330 | 110 | 50-450 | 130 | 20-2210 | 40 | 30-60 | 160 | 110-280 | 730 | 390-1920 | 140 | 130-180 | 300 | 130-1120 | 210 | 140-400 | 280 | 50-4330 | 110 | 80-210 | 160 | 130-220 | 50 | 40-70 |
| Female | 2011 | 250 | 120-850 | 250 | 180-430 | 180 | 60-950 |  |  | 50 | 30-120 | 220 | 160-380 | 190 | 100-500 | 170 | 140-220 | 170 | 90-550 | 230 | 150-460 | 110 | 20-2670 | 120 | 70-330 | 320 | 220-570 | 70 | 60-120 |
| Female | 2012 | 280 | 130-870 | 220 | 160-400 | 220 | 100-820 |  |  | 70 | 50-160 | 200 | 160-280 | 130 | 50-530 | 220 | 180-310 | 180 | 60-960 | 220 | 140-430 |  |  | 120 | 80-250 | 470 | 350-760 | 70 | 50-130 |
| Female | 2013 | 240 | 110-820 | 190 | 130-350 | 150 | 70-510 |  |  | 50 | 40-90 | 270 | 220-400 | 280 | 140-930 | 190 | 160-240 | 270 | 70-2150 | 210 | 140-420 |  |  | 240 | 170-460 | 500 | 400-720 | 60 | 50-100 |
| Female | 2014 | 440 | 150-2410 | 450 | 250-1130 | 530 | 170-3220 |  |  | 120 | 80-260 | 310 | 190-720 | 120 | 60-530 | 160 | 140-240 | 40 | 20-290 | 200 | 110-590 |  |  | 310 | 130-1250 | 240 | 190-370 | 70 | 50-140 |
| Male | 2001 |  |  |  |  |  |  |  |  |  |  |  |  |  |  |  |  |  |  |  |  |  |  |  |  |  |  |  |  |
| Male | 2002 |  |  | 210 | 160-340 | 430 | 250-1000 |  |  | 70 | 50-160 |  |  |  |  | 180 | 90-580 |  |  |  |  |  |  |  |  |  |  |  |  |
| Male | 2003 |  |  | 330 | 240-530 | 620 | 360-1440 |  |  | 70 | 60-120 |  |  | 330 | 230-620 | 240 | 180-400 |  |  | 510 | 170-2890 | 440 | 270-950 |  |  | 70 | 30-380 |  |  |
| Male | 2004 |  |  | 230 | 180-350 | 630 | 370-1440 |  |  | 70 | 60-110 |  |  | 280 | 190-530 | 200 | 170-270 |  |  | 120 | 70-330 | 430 | 300-770 |  |  | 80 | 40-300 |  |  |
| Male | 2005 |  |  | 290 | 210-470 | 570 | 390-1040 | 80 | 40-240 | 110 | 70-230 |  |  | 350 | 260-550 | 280 | 240-360 | 430 | 180-1900 | 110 | 60-290 | 680 | 400-1520 | 240 | 50-3640 | 140 | 60-480 | 110 | 70-230 |
| Male | 2006 |  |  | 500 | 350-850 | 620 | 450-1020 | 120 | 60-410 | 90 | 70-130 | 100 | 60-260 | 940 | 610-1780 | 290 | 260-350 | 1050 | 520-3100 | 290 | 140-940 | 800 | 470-1830 | 280 | 70-2540 | 170 | 70-720 | 170 | 80-630 |
| Male | 2007 |  |  | 290 | 230-420 | 790 | 570-1300 | 50 | 30-150 | 100 | 80-170 | 230 | 100-920 | 540 | 420-800 | 260 | 240-290 | 570 | 380-1100 | 200 | 130-430 | 520 | 340-1050 | 230 | 90-1140 | 580 | 280-1840 | 130 | 80-260 |
| Male | 2008 | 160 | 90-390 | 500 | 360-810 | 610 | 450-990 | 90 | 40-370 | 90 | 60-170 | 180 | 100-410 | 640 | 500-960 | 260 | 220-350 | 560 | 370-1100 | 290 | 220-480 | 360 | 210-870 | 350 | 110-2030 | 140 | 100-270 | 500 | 210-1850 |
| Male | 2009 | 120 | 80-240 | 300 | 240-420 | 1120 | 790-1910 | 40 | 20-200 | 80 | 60-160 | 200 | 130-410 | 530 | 430-740 | 290 | 240-380 | 600 | 400-1140 | 170 | 130-280 | 250 | 130-720 | 400 | 190-1250 | 130 | 100-200 | 150 | 100-320 |
| Male | 2010 | 150 | 80-450 | 500 | 390-760 | 1260 | 930-2010 |  |  | 60 | 40-100 | 320 | 240-530 | 470 | 360-690 | 250 | 210-360 | 380 | 280-630 | 180 | 120-340 | 400 | 150-1830 | 220 | 130-560 | 600 | 350-1380 | 100 | 70-190 |
| Male | 2011 | 160 | 80-420 | 420 | 320-620 | 1080 | 860-1540 |  |  | 90 | 60-160 | 190 | 150-260 | 390 | 290-620 | 200 | 160-280 | 610 | 410-1140 | 240 | 160-480 | 250 | 50-3340 | 90 | 60-170 | 330 | 220-630 | 120 | 70-230 |
| Male | 2012 | 440 | 190-1570 | 520 | 390-820 | 1420 | 1100-2120 |  |  | 80 | 60-130 | 230 | 190-320 | 370 | 250-700 | 170 | 130-250 | 580 | 380-1100 | 480 | 300-1010 |  |  | 230 | 140-520 | 390 | 270-690 | 100 | 70-190 |
| Male | 2013 | 200 | 120-490 | 340 | 270-480 | 1050 | 820-1560 |  |  | 80 | 60-110 | 220 | 170-330 | 370 | 250-670 | 210 | 150-380 | 320 | 200-630 | 210 | 140-400 |  |  | 200 | 130-460 | 550 | 380-1020 | 80 | 50-160 |
| Male | 2014 | 270 | 90-1550 | 480 | 340-800 | 890 | 670-1380 |  |  | 120 | 90-220 | 160 | 110-290 | 190 | 130-360 | 190 | 110-470 | 460 | 210-1600 | 230 | 130-610 |  |  | 170 | 60-1010 | 400 | 210-1180 | 110 | 60-280 |

**Table S7.** Estimated recapture probabilities, p, and associated 95% confidence intervals (CI) for adult female and male Lake Erie Watersnakes.

|  |  | Kelleys Island | | | | | | | | | | South Bass Island | | | | | | Middle Bass Island | | | | | | North Bass Island | | | | Gibraltar | |
| --- | --- | --- | --- | --- | --- | --- | --- | --- | --- | --- | --- | --- | --- | --- | --- | --- | --- | --- | --- | --- | --- | --- | --- | --- | --- | --- | --- | --- | --- |
|  |  | Long Point | | Southeast Shore | | South Shore | | Minshall | | State Park | | East Point | | East Shore | | State Park | | East Point | | State Park | | West End | | NE,E,SE Shore | | South Shore | |  |  |
| Sex | Year | p | CI | p | CI | P | CI | P | CI | P | CI | P | CI | P | CI | P | CI | P | CI | P | CI | P | CI | P | CI | P | CI | P | CI |
| Female | 2001 | 0.08 | 0.01-0.39 |  |  | 0.41 | 0.09-0.83 | 0.26 | 0.06-0.66 |  |  |  |  |  |  |  |  |  |  |  |  |  |  |  |  |  |  |  |  |
| Female | 2002 | 0.11 | 0.04-0.27 | 0.38 | 0.29-0.47 | 0.16 | 0.09-0.26 |  |  | 0.33 | 0.16-0.56 |  |  |  |  | 0.28 | 0.12-0.53 |  |  |  |  |  |  |  |  | 0.10 | 0.01-0.48 |  |  |
| Female | 2003 | 0.02 | 0.00-0.13 | 0.25 | 0.19-0.32 | 0.15 | 0.10-0.23 | 0.06 | 0.01-0.33 | 0.52 | 0.36-0.68 |  |  | 0.23 | 0.15-0.33 | 0.58 | 0.45-0.70 |  |  | 0.36 | 0.18-0.6 | 0.12 | 0.07-0.20 |  |  | 0.28 | 0.15-0.45 |  |  |
| Female | 2004 | 0.05 | 0.01-0.17 | 0.33 | 0.26-0.41 | 0.07 | 0.04-0.13 | 0.34 | 0.13-0.64 | 0.51 | 0.38-0.64 |  |  | 0.12 | 0.07-0.19 | 0.50 | 0.42-0.58 |  |  | 0.24 | 0.14-0.38 | 0.19 | 0.14-0.27 |  |  | 0.23 | 0.14-0.37 | 0.26 | 0.12-0.48 |
| Female | 2005 |  |  | 0.19 | 0.14-0.25 | 0.14 | 0.09-0.21 | 0.15 | 0.06-0.32 | 0.23 | 0.14-0.34 |  |  | 0.25 | 0.18-0.33 | 0.61 | 0.53-0.69 | 0.11 | 0.04-0.30 | 0.11 | 0.06-0.21 | 0.14 | 0.10-0.20 | 0.05 | 0.01-0.26 | 0.26 | 0.17-0.37 | 0.38 | 0.26-0.52 |
| Female | 2006 | 0.08 | 0.03-0.23 | 0.18 | 0.13-0.24 | 0.17 | 0.12-0.24 | 0.26 | 0.13-0.45 | 0.66 | 0.52-0.78 | 0.27 | 0.15-0.45 | 0.15 | 0.10-0.21 | 0.58 | 0.51-0.64 | 0.08 | 0.04-0.17 | 0.18 | 0.1-0.3 | 0.10 | 0.06-0.15 | 0.09 | 0.03-0.22 | 0.10 | 0.05-0.18 | 0.22 | 0.14-0.34 |
| Female | 2007 | 0.23 | 0.13-0.39 | 0.28 | 0.22-0.35 | 0.17 | 0.12-0.23 | 0.17 | 0.07-0.35 | 0.38 | 0.27-0.49 | 0.12 | 0.05-0.26 | 0.20 | 0.15-0.26 | 0.79 | 0.73-0.85 | 0.18 | 0.12-0.27 | 0.25 | 0.16-0.37 | 0.12 | 0.08-0.17 | 0.31 | 0.20-0.46 | 0.24 | 0.15-0.35 | 0.41 | 0.29-0.55 |
| Female | 2008 | 0.28 | 0.19-0.41 | 0.20 | 0.15-0.25 | 0.14 | 0.10-0.20 | 0.12 | 0.04-0.31 | 0.37 | 0.26-0.49 | 0.13 | 0.07-0.24 | 0.19 | 0.15-0.25 | 0.38 | 0.32-0.44 | 0.10 | 0.07-0.16 | 0.62 | 0.52-0.71 | 0.11 | 0.08-0.17 | 0.10 | 0.05-0.18 | 0.31 | 0.24-0.40 | 0.06 | 0.02-0.13 |
| Female | 2009 | 0.26 | 0.19-0.36 | 0.24 | 0.18-0.30 | 0.14 | 0.10-0.02 | 0.37 | 0.18-0.62 | 0.30 | 0.19-0.43 | 0.20 | 0.12-0.30 | 0.26 | 0.21-0.32 | 0.51 | 0.43-0.58 | 0.14 | 0.09-0.20 | 0.35 | 0.28-0.43 | 0.05 | 0.03-0.08 | 0.23 | 0.15-0.34 | 0.34 | 0.26-0.43 | 0.41 | 0.30-0.53 |
| Female | 2010 | 0.17 | 0.11-0.25 | 0.32 | 0.26-0.39 | 0.16 | 0.12-0.22 | 0.03 | 0.00-0.22 | 0.43 | 0.30-0.58 | 0.42 | 0.32-0.52 | 0.21 | 0.16-0.26 | 0.44 | 0.37-0.51 | 0.15 | 0.10-0.21 | 0.21 | 0.16-0.28 | 0.05 | 0.03-0.09 | 0.30 | 0.21-0.42 | 0.31 | 0.23-0.39 | 0.36 | 0.25-0.47 |
| Female | 2011 | 0.13 | 0.08-0.21 | 0.26 | 0.21-0.32 | 0.17 | 0.13-0.23 | 0.25 | 0.09-0.52 | 0.45 | 0.31-0.59 | 0.33 | 0.25-0.42 | 0.14 | 0.10-0.18 | 0.42 | 0.35-0.49 | 0.17 | 0.12-0.24 | 0.23 | 0.16-0.31 | 0.03 | 0.01-0.06 | 0.16 | 0.10-0.25 | 0.20 | 0.14-0.27 | 0.36 | 0.26-0.48 |
| Female | 2012 | 0.09 | 0.05-0.16 | 0.21 | 0.16-0.27 | 0.17 | 0.13-0.22 |  |  | 0.39 | 0.26-0.53 | 0.44 | 0.36-0.53 | 0.11 | 0.07-0.15 | 0.40 | 0.32-0.48 | 0.14 | 0.09-0.20 | 0.24 | 0.18-0.33 | 0.01 | 0.00-0.04 | 0.25 | 0.16-0.36 | 0.27 | 0.20-0.34 | 0.45 | 0.34-0.58 |
| Female | 2013 | 0.14 | 0.09-0.21 | 0.28 | 0.22-0.35 | 0.20 | 0.16-0.25 |  |  | 0.62 | 0.47-0.75 | 0.51 | 0.42-0.59 | 0.18 | 0.13-0.24 | 0.54 | 0.45-0.62 | 0.15 | 0.10-0.21 | 0.24 | 0.17-0.32 |  |  | 0.49 | 0.36-0.61 | 0.48 | 0.41-0.56 | 0.44 | 0.32-0.57 |
| Female | 2014 | 0.11 | 0.07-0.18 | 0.18 | 0.13-0.24 | 0.21 | 0.16-0.26 |  |  | 0.50 | 0.38-0.63 | 0.29 | 0.23-0.36 | 0.14 | 0.10-0.20 | 0.43 | 0.35-0.51 | 0.11 | 0.07-0.17 | 0.30 | 0.22-0.4 |  |  | 0.15 | 0.10-0.21 | 0.25 | 0.20-0.30 | 0.35 | 0.24-0.48 |
| Male | 2001 | 0.06 | 0.01-0.34 |  |  | 0.36 | 0.08-0.80 | 0.22 | 0.05-0.61 |  |  |  |  |  |  |  |  |  |  |  |  |  |  |  |  |  |  |  |  |
| Male | 2002 | 0.10 | 0.04-0.23 | 0.33 | 0.25-0.42 | 0.13 | 0.08-0.22 |  |  | 0.28 | 0.13-0.50 |  |  |  |  | 0.24 | 0.01-0.48 |  |  |  |  |  |  |  |  | 0.09 | 0.01-0.43 |  |  |
| Male | 2003 | 0.02 | 0.00-0.11 | 0.21 | 0.16-0.28 | 0.13 | 0.08-0.19 | 0.05 | 0.01-0.28 | 0.47 | 0.32-0.63 |  |  | 0.19 | 0.13-0.28 | 0.53 | 0.40-0.65 |  |  | 0.32 | 0.15-0.55 | 0.10 | 0.05-0.17 |  |  | 0.24 | 0.13-0.40 |  |  |
| Male | 2004 | 0.04 | 0.01-0.14 | 0.29 | 0.23-0.36 | 0.06 | 0.03-0.10 | 0.30 | 0.11-0.59 | 0.46 | 0.33-0.59 |  |  | 0.10 | 0.06-0.16 | 0.45 | 0.37-0.53 |  |  | 0.20 | 0.11-0.33 | 0.16 | 0.11-0.23 |  |  | 0.20 | 0.11-0.32 | 0.22 | 0.10-0.43 |
| Male | 2005 |  |  | 0.16 | 0.12-0.22 | 0.12 | 0.08-0.18 | 0.12 | 0.05-0.27 | 0.19 | 0.12-0.30 |  |  | 0.21 | 0.15-0.29 | 0.56 | 0.48-0.64 | 0.09 | 0.03-0.26 | 0.09 | 0.05-0.18 | 0.12 | 0.08-0.17 | 0.04 | 0.01-0.22 | 0.22 | 0.14-0.33 | 0.33 | 0.22-0.47 |
| Male | 2006 | 0.07 | 0.02-0.19 | 0.15 | 0.11-0.21 | 0.14 | 0.10-0.20 | 0.22 | 0.11-0.40 | 0.61 | 0.47-0.74 | 0.24 | 0.12-0.40 | 0.12 | 0.08-0.18 | 0.53 | 0.46-0.59 | 0.07 | 0.03-0.14 | 0.15 | 0.09-0.26 | 0.08 | 0.05-0.12 | 0.07 | 0.03-0.18 | 0.08 | 0.04-0.15 | 0.19 | 0.11-0.29 |
| Male | 2007 | 0.20 | 0.10-0.34 | 0.24 | 0.19-0.30 | 0.14 | 0.10-0.19 | 0.14 | 0.06-0.30 | 0.33 | 0.23-0.44 | 0.10 | 0.04-0.22 | 0.17 | 0.13-0.22 | 0.76 | 0.68-0.82 | 0.15 | 0.10-0.23 | 0.21 | 0.13-0.32 | 0.10 | 0.06-0.14 | 0.27 | 0.16-0.41 | 0.20 | 0.13-0.31 | 0.36 | 0.25-0.49 |
| Male | 2008 | 0.24 | 0.16-0.36 | 0.16 | 0.12-0.22 | 0.12 | 0.08-0.16 | 0.10 | 0.04-0.26 | 0.32 | 0.22-0.44 | 0.11 | 0.06-0.20 | 0.16 | 0.13-0.21 | 0.33 | 0.27-0.39 | 0.09 | 0.05-0.14 | 0.57 | 0.47-0.67 | 0.09 | 0.06-0.14 | 0.08 | 0.04-0.15 | 0.27 | 0.20-0.35 | 0.05 | 0.02-0.11 |
| Male | 2009 | 0.23 | 0.16-0.32 | 0.20 | 0.15-0.26 | 0.12 | 0.08-0.16 | 0.32 | 0.15-0.57 | 0.25 | 0.16-0.38 | 0.16 | 0.10-0.26 | 0.22 | 0.18-0.27 | 0.45 | 0.38-0.53 | 0.11 | 0.08-0.17 | 0.31 | 0.24-0.38 | 0.04 | 0.02-0.07 | 0.19 | 0.12-0.29 | 0.29 | 0.22-0.38 | 0.36 | 0.26-0.48 |
| Male | 2010 | 0.14 | 0.09-0.21 | 0.27 | 0.22-0.34 | 0.13 | 0.10-0.18 | 0.03 | 0.00-0.18 | 0.38 | 0.26-0.52 | 0.37 | 0.28-0.47 | 0.17 | 0.14-0.22 | 0.39 | 0.32-0.46 | 0.12 | 0.09-0.18 | 0.18 | 0.13-0.24 | 0.04 | 0.02-0.07 | 0.26 | 0.17-0.37 | 0.26 | 0.20-0.34 | 0.31 | 0.22-0.42 |
| Male | 2011 | 0.11 | 0.07-0.17 | 0.22 | 0.18-0.28 | 0.15 | 0.11-0.19 | 0.21 | 0.08-0.47 | 0.40 | 0.27-0.54 | 0.29 | 0.22-0.37 | 0.11 | 0.08-0.15 | 0.37 | 0.30-0.44 | 0.14 | 0.10-0.20 | 0.19 | 0.14-0.27 | 0.02 | 0.01-0.05 | 0.14 | 0.08-0.21 | 0.17 | 0.12-0.23 | 0.31 | 0.22-0.42 |
| Male | 2012 | 0.07 | 0.04-0.13 | 0.18 | 0.14-0.23 | 0.14 | 0.11-0.18 |  |  | 0.34 | 0.22-0.48 | 0.39 | 0.31-0.47 | 0.09 | 0.06-0.12 | 0.35 | 0.28-0.43 | 0.11 | 0.08-0.16 | 0.21 | 0.15-0.29 | 0.01 | 0.00-0.03 | 0.21 | 0.13-0.31 | 0.23 | 0.17-0.30 | 0.40 | 0.29-0.53 |
| Male | 2013 | 0.12 | 0.07-0.18 | 0.24 | 0.19-0.30 | 0.17 | 0.14-0.21 |  |  | 0.57 | 0.42-0.70 | 0.46 | 0.37-0.54 | 0.15 | 0.11-0.20 | 0.48 | 0.40-0.57 | 0.12 | 0.08-0.18 | 0.20 | 0.14-0.28 |  |  | 0.43 | 0.32-0.56 | 0.43 | 0.36-0.51 | 0.39 | 0.28-0.52 |
| Male | 2014 | 0.09 | 0.06-0.15 | 0.15 | 0.11-0.20 | 0.17 | 0.14-0.22 |  |  | 0.45 | 0.33-0.58 | 0.25 | 0.19-0.32 | 0.12 | 0.08-0.17 | 0.38 | 0.30-0.46 | 0.09 | 0.06-0.14 | 0.26 | 0.19-0.35 |  |  | 0.12 | 0.08-0.18 | 0.21 | 0.17-0.26 | 0.31 | 0.20-0.43 |

**Table S8.** Estimated realized population growth rates, λ, and associated 95% confidence intervals (CI) for adult female and male Lake Erie Watersnakes.

|  |  | Kelleys Island | | | | | | | | | | South Bass Island | | | | | | Middle Bass Island | | | | | | North Bass Island | | | | Gibraltar | |
| --- | --- | --- | --- | --- | --- | --- | --- | --- | --- | --- | --- | --- | --- | --- | --- | --- | --- | --- | --- | --- | --- | --- | --- | --- | --- | --- | --- | --- | --- |
|  |  | Long Point | | Southeast Shore | | South Shore | | Minshall | | State Park | | East Point | | East Shore | | State Park | | East Point | | State Park | | West End | | NE,E,SE Shore | | South Shore | |  |  |
| Sex | Year | λ | CI | λ | CI | λ | CI | λ | CI | λ | CI | λ | CI | λ | CI | λ | CI | λ | CI | λ | CI | λ | CI | λ | CI | λ | CI | λ | CI |
| Female | 2002 | 1.09 | 0.88-1.35 | 1.04 | 0.84-1.29 | 1.01 | 0.81-1.25 | 0.97 | 0.77-1.24 | 1.08 | 0.87-1.34 |  |  | 1.04 | 0.84-1.30 | 1.05 | 0.84-1.30 |  |  | 1.04 | 0.84-1.30 | 1.09 | 0.86-1.37 |  |  | 1.15 | 0.93-1.44 |  |  |
| Female | 2003 | 1.03 | 0.89-1.19 | 0.98 | 0.85-1.13 | 0.95 | 0.83-1.09 | 0.92 | 0.77-1.10 | 1.02 | 0.89-1.17 |  |  | 0.99 | 0.86-1.14 | 0.99 | 0.86-1.14 | 0.93 | 0.80-1.08 | 0.99 | 0.85-1.14 | 1.03 | 0.88-1.20 |  |  | 1.09 | 0.95-1.26 | 1.02 | 0.88-1.18 |
| Female | 2004 | 1.24 | 1.10-1.39 | 1.18 | 1.05-1.32 | 1.14 | 1.01-1.28 | 1.10 | 0.94-1.29 | 1.22 | 1.09-1.37 |  |  | 1.18 | 1.05-1.33 | 1.18 | 1.06-1.33 | 1.11 | 0.98-1.26 | 1.18 | 1.05-1.33 | 1.23 | 1.08-1.40 | 1.24 | 1.10-1.41 | 1.31 | 1.17-1.47 | 1.22 | 1.08-1.37 |
| Female | 2005 | 1.15 | 1.03-1.28 | 1.09 | 0.99-1.21 | 1.06 | 0.95-1.18 | 1.03 | 0.88-1.19 | 1.14 | 1.02-1.26 | 1.20 | 1.07-1.35 | 1.10 | 0.99-1.22 | 1.10 | 0.99-1.22 | 1.03 | 0.92-1.16 | 1.10 | 0.99-1.22 | 1.14 | 1.01-1.29 | 1.16 | 1.03-1.29 | 1.22 | 1.10-1.35 | 1.14 | 1.02-1.26 |
| Female | 2006 | 1.01 | 0.92-1.11 | 0.96 | 0.88-1.05 | 0.93 | 0.85-1.02 | 0.90 | 0.79-1.04 | 1.00 | 0.92-1.09 | 1.06 | 0.96-1.17 | 0.97 | 0.88-1.06 | 0.97 | 0.89-1.06 | 0.91 | 0.82-1.01 | 0.97 | 0.88-1.06 | 1.01 | 0.90-1.12 | 1.02 | 0.92-1.12 | 1.07 | 0.98-1.17 | 1.00 | 0.91-1.10 |
| Female | 2007 | 1.02 | 0.94-1.12 | 0.97 | 0.90-1.05 | 0.94 | 0.87-1.02 | 0.91 | 0.80-1.05 | 1.01 | 0.93-1.10 | 1.07 | 0.98-1.17 | 0.98 | 0.90-1.07 | 0.98 | 0.91-1.06 | 0.92 | 0.84-1.01 | 0.98 | 0.90-1.07 | 1.02 | 0.92-1.13 | 1.03 | 0.94-1.13 | 1.08 | 1.00-1.18 | 1.01 | 0.93-1.10 |
| Female | 2008 | 1.06 | 0.97-1.16 | 1.01 | 0.93-1.09 | 0.98 | 0.90-1.06 | 0.94 | 0.82-1.09 | 1.05 | 0.96-1.14 | 1.11 | 1.01-1.22 | 1.01 | 0.93-1.10 | 1.01 | 0.93-1.10 | 0.95 | 0.86-1.05 | 1.01 | 0.93-1.10 | 1.05 | 0.95-1.17 | 1.06 | 0.97-1.17 | 1.12 | 1.03-1.22 | 1.05 | 0.96-1.14 |
| Female | 2009 | 0.99 | 0.9-1.08 | 0.94 | 0.87-1.02 | 0.91 | 0.84-0.99 | 0.88 | 0.77-1.01 | 0.98 | 0.90-1.06 | 1.03 | 0.94-1.13 | 0.94 | 0.87-1.03 | 0.95 | 0.87-1.02 | 0.89 | 0.81-0.98 | 0.94 | 0.87-1.03 | 0.98 | 0.88-1.09 | 0.99 | 0.90-1.09 | 1.04 | 0.96-1.13 | 0.97 | 0.89-1.06 |
| Female | 2010 | 1.09 | 0.99-1.19 | 1.03 | 0.95-1.12 | 1.00 | 0.92-1.09 | 0.97 | 0.84-1.11 | 1.07 | 0.98-1.17 | 1.13 | 1.03-1.25 | 1.04 | 0.95-1.13 | 1.04 | 0.96-1.13 | 0.97 | 0.88-1.08 | 1.04 | 0.95-1.13 | 1.08 | 0.97-1.20 | 1.09 | 0.99-1.20 | 1.15 | 1.05-1.25 | 1.07 | 0.98-1.17 |
| Female | 2011 | 1.10 | 1.00-1.22 | 1.05 | 0.96-1.15 | 1.02 | 0.93-1.11 | 0.99 | 0.85-1.14 | 1.09 | 1.00-1.20 | 1.15 | 1.04-1.27 | 1.06 | 0.96-1.16 | 1.06 | 0.97-1.16 | 0.99 | 0.89-1.10 | 1.06 | 0.96-1.16 | 1.10 | 0.98-1.23 | 1.11 | 1.01-1.23 | 1.17 | 1.07-1.28 | 1.09 | 0.99-1.20 |
| Female | 2012 | 0.97 | 0.89-1.06 | 0.92 | 0.85-1.00 | 0.89 | 0.82-0.97 |  |  | 0.96 | 0.88-1.04 | 1.01 | 0.92-1.11 | 0.93 | 0.85-1.01 | 0.93 | 0.86-1.01 | 0.87 | 0.79-0.96 | 0.92 | 0.85-1.01 | 0.96 | 0.87-1.07 | 0.97 | 0.89-1.07 | 1.02 | 0.94-1.11 | 0.96 | 0.88-1.04 |
| Female | 2013 | 1.10 | 1.01-1.20 | 1.05 | 0.97-1.13 | 1.01 | 0.94-1.09 |  |  | 1.09 | 1.01-1.18 | 1.15 | 1.05-1.25 | 1.05 | 0.97-1.14 | 1.05 | 0.98-1.14 | 0.99 | 0.90-1.08 | 1.05 | 0.97-1.14 | 1.09 | 0.99-1.21 | 1.11 | 1.01-1.21 | 1.16 | 1.08-1.26 | 1.09 | 1.00-1.18 |
| Male | 2002 | 1.14 | 0.92-1.42 | 1.05 | 0.85-1.30 | 1.08 | 0.87-1.34 | 0.97 | 0.76-1.23 | 1.08 | 0.87-1.34 |  |  | 1.02 | 0.82-1.27 | 1.00 | 0.80-1.24 |  |  | 1.03 | 0.83-1.28 | 1.14 | 0.90-1.43 |  |  | 1.18 | 0.95-1.47 | 1.00 | 0.87-1.16 |
| Male | 2003 | 1.08 | 0.93-1.25 | 0.99 | 0.86-1.14 | 1.02 | 0.89-1.17 | 0.92 | 0.77-1.09 | 1.02 | 0.88-1.17 |  |  | 0.96 | 0.84-1.11 | 0.94 | 0.82-1.08 | 0.94 | 0.82-1.09 | 0.97 | 0.84-1.12 | 1.07 | 0.92-1.25 |  |  | 1.12 | 0.97-1.28 | 1.20 | 1.07-1.35 |
| Male | 2004 | 1.30 | 1.15-1.46 | 1.19 | 1.06-1.33 | 1.22 | 1.09-1.37 | 1.10 | 0.94-1.28 | 1.22 | 1.09-1.36 |  |  | 1.15 | 1.03-1.30 | 1.13 | 1.01-1.27 | 1.13 | 1.00-1.28 | 1.16 | 1.03-1.31 | 1.29 | 1.13-1.46 | 1.21 | 1.06-1.37 | 1.34 | 1.19-1.50 | 1.12 | 1.00-1.24 |
| Male | 2005 | 1.21 | 1.08-1.35 | 1.11 | 1.00-1.23 | 1.14 | 1.03-1.26 | 1.02 | 0.88-1.18 | 1.13 | 1.02-1.26 | 1.10 | 0.98-1.23 | 1.07 | 0.97-1.19 | 1.05 | 0.95-1.16 | 1.05 | 0.94-1.18 | 1.08 | 0.97-1.21 | 1.20 | 1.06-1.35 | 1.12 | 1.00-1.26 | 1.24 | 1.12-1.38 | 0.98 | 0.90-1.08 |
| Male | 2006 | 1.06 | 0.96-1.17 | 0.97 | 0.89-1.06 | 1.00 | 0.92-1.09 | 0.90 | 0.79-1.03 | 1.00 | 0.91-1.09 | 0.97 | 0.88-1.07 | 0.94 | 0.86-1.03 | 0.92 | 0.85-1.01 | 0.93 | 0.84-1.02 | 0.95 | 0.87-1.04 | 1.05 | 0.95-1.17 | 0.99 | 0.89-1.09 | 1.09 | 1.00-1.20 | 0.99 | 0.91-1.08 |
| Male | 2007 | 1.07 | 0.98-1.18 | 0.98 | 0.91-1.07 | 1.02 | 0.94-1.10 | 0.91 | 0.80-1.04 | 1.01 | 0.93-1.10 | 0.98 | 0.89-1.07 | 0.96 | 0.88-1.04 | 0.94 | 0.87-1.01 | 0.94 | 0.86-1.03 | 0.96 | 0.88-1.05 | 1.07 | 0.96-1.18 | 1.00 | 0.91-1.10 | 1.11 | 1.02-1.20 | 1.03 | 0.94-1.12 |
| Male | 2008 | 1.11 | 1.01-1.22 | 1.02 | 0.94-1.10 | 1.05 | 0.97-1.14 | 0.94 | 0.82-1.07 | 1.04 | 0.96-1.13 | 1.01 | 0.92-1.11 | 0.99 | 0.91-1.08 | 0.97 | 0.89-1.05 | 0.97 | 0.88-1.06 | 1.00 | 0.91-1.09 | 1.10 | 0.99-1.22 | 1.03 | 0.94-1.14 | 1.15 | 1.05-1.25 | 0.96 | 0.88-1.05 |
| Male | 2009 | 1.03 | 0.94-1.13 | 0.95 | 0.88-1.03 | 0.98 | 0.90-1.06 | 0.88 | 0.77-1.00 | 0.97 | 0.90-1.06 | 0.94 | 0.86-1.04 | 0.92 | 0.85-1.00 | 0.90 | 0.83-0.98 | 0.90 | 0.82-0.99 | 0.93 | 0.85-1.01 | 1.03 | 0.93-1.14 | 0.96 | 0.88-1.06 | 1.07 | 0.98-1.16 | 1.05 | 0.96-1.15 |
| Male | 2010 | 1.14 | 1.03-1.25 | 1.04 | 0.96-1.14 | 1.08 | 0.99-1.17 | 0.96 | 0.84-1.10 | 1.07 | 0.98-1.17 | 1.04 | 0.94-1.14 | 1.01 | 0.93-1.11 | 0.99 | 0.91-1.08 | 0.99 | 0.90-1.09 | 1.02 | 0.93-1.12 | 1.13 | 1.02-1.26 | 1.06 | 0.96-1.17 | 1.17 | 1.08-1.28 | 1.07 | 0.98-1.18 |
| Male | 2011 | 1.16 | 1.05-1.28 | 1.06 | 0.97-1.16 | 1.09 | 1.00-1.20 | 0.98 | 0.85-1.13 | 1.09 | 0.99-1.19 | 1.06 | 0.96-1.16 | 1.03 | 0.94-1.13 | 1.01 | 0.92-1.10 | 1.01 | 0.92-1.12 | 1.04 | 0.95-1.14 | 1.15 | 1.03-1.28 | 1.08 | 0.97-1.19 | 1.19 | 1.09-1.31 | 0.94 | 0.86-1.03 |
| Male | 2012 | 1.01 | 0.93-1.11 | 0.93 | 0.86-1.01 | 0.96 | 0.88-1.04 |  |  | 0.95 | 0.88-1.04 | 0.93 | 0.85-1.01 | 0.90 | 0.83-0.98 | 0.88 | 0.82-0.96 | 0.89 | 0.81-0.97 | 0.91 | 0.84-0.99 | 1.01 | 0.91-1.12 | 0.94 | 0.86-1.04 | 1.05 | 0.96-1.14 | 1.07 | 0.98-1.16 |
| Male | 2013 | 1.15 | 1.06-1.26 | 1.06 | 0.98-1.14 | 1.09 | 1.01-1.18 |  |  | 1.08 | 1.00-1.17 | 1.05 | 0.96-1.15 | 1.03 | 0.95-1.11 | 1.01 | 0.93-1.08 | 1.01 | 0.92-1.10 | 1.04 | 0.95-1.12 | 1.14 | 1.04-1.26 | 1.07 | 0.98-1.17 | 1.19 | 1.10-1.29 | 1.00 | 0.87-1.16 |

**File S1.** Excel file containing estimates of annual survival among snake taxa. The file consists to a Data sheet which includes details for each study and an Abbreviations sheet which provides definitions of abbreviations. Citations included in this file are listed below.

Altwegg, R., Dummermuth, S., Anholt, B. R. & Flatt, T. (2005). Winter weather affects asp viper *Vipera aspis* population dynamics through susceptible juveniles. *Oikos,* **110,** 55-66.

Baker, S. J. (2016). Life and death in a corn desert oasis: reproduction, mortality, genetic diversity, and viability of Illinois’ last eastern massasauga population. PhD thesis, University of Illinois, Urban-Champaign.

Baron, J. P., Le Galliard, J. F., Ferriere, R. & Tully, T. (2013). Intermittent breeding and the dynamics of resource allocation to reproduction, growth and survival. *Functional Ecology,* **27,** 173-183.

Baron, J. P., Le Galliard, J. F., Tully, T. & Ferriere, R. (2010). Cohort variation in offspring growth and survival: prenatal and postnatal factors in a late-maturing viviparous snake. *Journal of Animal Ecology,* **79,** 640-649.

Bonnet, X., Lorioux, S., Pearson, D., Aubret, F., Bradshaw, D., Delmas, V. & Fauvel, T. (2011). Which proximate factor determines sexual size dimorphism in tiger snakes? *Biological Journal of the Linnean Society,* **103,** 668-680.

Breininger, D. R., Mazerolle, M. J., Bolt, M. R., Legare, M. L., Drese, J. H. & Hines, J. E. (2012). Habitat fragmentation effects on annual survival of the federally protected eastern indigo snake. *Animal Conservation,* **15,** 361-368.

Bronikowski, A. & Vleck, D. (2010). Metabolism, body size and life span: a case study in evolutionarily divergent populations of the garter snake (*Thamnophis elegans*). *Integrative and Comparative Biology,* **50,** 880-887.

Brown, G. P. & Shine, R. (2002). Reproductive ecology of a tropical natricine snake, *Tropidonophis mairii* (Colubridae). *Journal of Zoology,* **258,** 63-72.

Brown, G. P., Shine, R. & Madsen, T. (2002). Responses of three sympatric snake species to tropical seasonality in northern Australia. *Journal of Tropical Ecology,* **18,** 549-568.

Brown, G. P., Ujvari, B., Madsen, T. & Shine, R. (2013). Invader impact clarifies the roles of top-down and bottom-up effects on tropical snake populations. *Functional Ecology,* **27,** 351-361.

Brown, G. P. & Weatherhead, P. J. (1999). Demography and sexual size dimorphism in northern water snakes, *Nerodia sipedon*. *Canadian Journal of Zoology-Revue Canadienne De Zoologie,* **77,** 1358-1366.

Brown, W. S. (1991). Female reproductive ecology in a northern population of the timber rattlesnake, *Crotalus horridus*. *Herpetologica,* **47,** 101-115.

Brown, W. S. (1993). Biology, status, and management of the Timber Rattlesnake (*Crotalus horridus*): a guide for conservation. *Society for the Study of Amphibians and Reptiles Herpetological Circulars,* **22,** 1-78.

Brown, W. S. (2008). Sampling Timber Rattlesnakes (*Crotalus horridus*): phenology, growth, intimidation, survival, and a syndrome of undetermined origin in a northern population. *The Biology of Rattlesnakes* (eds W. K. Hayes, K. R. Beaman, M. D. Cardwell & S. P. Buse), pp. 235-256. Loma Linda University Press, Loma Linda, CA.

Brown, W. S., Kery, M. & Hines, J. E. (2007). Survival of timber rattlesnakes (*Crotalus horridus*) estimated by capture-recapture models in relation to age, sex, color morph, time, and birthplace. *Copeia***,** 656-671.

Capula, M., Filippi, E., Luiselli, L. & Jesus, V. T. (1997). The ecology of the western whip snake, *Coluber viridiflaus* (Lacepede, 1789) in Mediterranean central Italy (Squamata: Serpentes: Colubridae). *Herpetozoa,* **10,** 65-79.

Cecala, K., Price, S. & Dorcas, M. (2010). Ecology of juvenile Northern watersnakes (*Nerodia sipedon*) inhabiting low-order streams. *Amphibia-Reptilia,* **31,** 169-174.

Chaitae, A. (2011). Demography of the Monocled Cobra (*Naja kaouthia*) in the central region of Thailand. MS thesis, University of Louisville, Louisville, KY.

Conant, R. (1975). *A Field Guide to Reptiles and Amphibians of Eastern and Central North America,* Houghton Mifflin Company, Boston.

Devan-Song, E. A. (2014). Ecology and conservation of the Bamboo Pit Viper: natural history, demography and effects of translocation. MS thesis, University of Rhode Island, Kingston, RI.

Diller, L. V. & Wallace, R. L. (1996). Comparative ecology of two snake species *(Crotalus viridis* and *Pituophis melanoleucus*) in Southwestern Idaho. *Herpetologica,* **52,** 343-360.

Diller, L. V. & Wallace, R. L. (2002). Growth, reproduction, and survival in a population of *Crotalus viridis oreganus* in north central Idaho. *Herpetological Monographs,* **16,** 26-45.

Dubey, S., Brown, G. P., Madsen, T. & Shine, R. (2009). Sexual selection favours large body size in males of a tropical snake (*Stegonotus cucullatus*, Colubridae). *Animal Behaviour,* **77,** 177-182.

Ernst, C. H. & Ernst, E. M. (2003). *Snakes of the United States and Canada,* Smithsonian Institution Press.

Fitch, H. S. (1999). *A Kansas Snake Community: Composition and Changes over 50 Years,* Krieger Publishing Company, Malabar, Florida.

Flatt, T., Dummermuth, S. & Anholt, B. R. (1997). Mark-recapture estimates of survival in populations of the asp viper, *Vipera aspis aspis*. *Journal of Herpetology,* **31,** 558-564.

Ford, N. B. (2002). Ecology of the Western Cottonmouth (*Agkistrodon piscivorus leucostoma*) in Northeastern Texas. *Biology of the Vipers* (eds G. W. Schuett, M. Hoggren, M. E. Douglas & H. W. Greene), pp. 167-178. Eagle Mountain Publishing, LC, Eagle Mountain, Utah.

Fornasiero, S., Bonnet, X., Dendi, F. & Zuffi, M. A. L. (2016). Growth, longevity and age at maturity in the European whip snakes, *Hierophis viridiflavus* and *H. carbonarius*. *Acta Herpetologica,* **11,** 135-149.

Forsman, A. (1993). Survival in relation to body size and growth rate in the adder, *Vipera berus*. *Journal of Animal Ecology,* **62,** 647-655.

Forsman, A. (1995). Opposing fitness consequences of color pattern in male and female snakes. *Journal of Evolutionary Biology,* **8,** 53-70.

Forsman, A. & Lindell, L. E. (1997). Responses of a predator to variation in prey abundance: Survival and emigration of adders in relation to vole density. *Canadian Journal of Zoology,* **75,** 1099-1108.

Gibbons, J. W. & Dorcas, M. (2004). *North American Watersnakes A Natural History,* University of Oklahoma Press, Norman.

Govindarajulu, P., Isaac, L. A., Engelstoft, C. & Ovaska, K. (2011). Relevance of life-history parameter estimation to conservation listing: case of the Sharp-tailed Snake (*Contia tenuis*). *Journal of Herpetology,* **45,** 300-307.

Greene, B. D., Dixon, J. R., Whiting, M. J. & Mueller, J. M. (1999). Reproductive ecology of the Concho water snake, *Nerodia harteri paucimaculata*. *Copeia***,** 701-709.

Gregory, P. T. (2004). Sexual dimorphism and allometric size variation in a population of Grass Snakes (*Natrix natrix*) in Southern England. *Journal of Herpetology,* **38,** 231-240.

Guimaraes, M., Munguia-Steyer, R., Doherty, P. F., Martins, M. & Sawaya, R. J. (2014). Population dynamics of the critically endangered Golden Lancehead pitviper, *Bothrops insularis*: stability or decline? *Plos One,* **9,** e95203. doi:10.1371/journal.pone.0095203.

Halstead, B. J., Wylie, G. D., Amarello, M., Smith, J. J., Thompson, M. E., Routman, E. J. & Casazza, M. L. (2011). Demography of the San Francisco Gartersnake in coastal San Mateo County, California. *Journal of Fish and Wildlife Management,* **2,** 41-48.

Halstead, B. J., Wylie, G. D., Coates, P. S., Valcarcel, P. & Casazza, M. L. (2012). Bayesian shared frailty models for regional inference about wildlife survival. *Animal Conservation,* **15,** 117-124.

Hansen, E. C., Scherer, R. D., White, G. C., Dickson, B. G. & Fleishman, E. (2015). Estimates of survival probability from two populations of Giant Gartersnakes in California's great central valley. *Copeia,* **103,** 1026-1036.

Hartmann, M. T., Marques, O. A. & Almeida-Santos, S. M. (2004). Reproductive biology of the southern Brazilian pitviper *Bothrops neuwiedi pubescens* (Serpentes, Viperidae). *Amphibia-Reptilia,* **25,** 77-85.

Hileman, E. T. (2016). Filling in the gaps in demography, phenology, and life history of the Eastern Massasauga Rattlesnake (*Sistrurus catenatus*). *Biological Sciences*, pp. 147. Northern Illinois University, DeKalb, IL.

Hileman, E. T., Kapfer, J. M., Muehlfeld, T. C. & Giovanni, J. H. (2015). Recouping lost information when mark-recapture data are pooled: a case study of Milksnakes (*Lampropeltis triangulum*) in the upper mdwestern United States. *Journal of Herpetology,* **49,** 428-436.

Hileman, E. T., Powell, R., Perry, G., Mougey, K., Thomas, R. & Henderson, R. W. (2017). Demography of the racer *Borikinophis portoricensis* (Squamata: Dipsadidae) on Guana Island, British Virgin Islands. *Journal of Herpetology*, **51,** 454-460.

Hyslop, N. L., Meyers, J. M., Cooper, R. J. & Norton, T. M. (2009). Survival of radio-implanted *Drymarchon couperi* (Eastern Indigo Snake) in relation to body size and sex. *Herpetologica,* **65,** 199-206.

Hyslop, N. L., Stevenson, D. J., Macey, J. N., Carlile, L. D., Jenkins, C. L., Hostetler, J. A. & Oli, M. K. (2012). Survival and population growth of a long-lived threatened snake species, *Drymarchon couperi* (Eastern Indigo Snake). *Population Ecology,* **54,** 145-156.

Johnson, B. D. (2013). Management and status of an endangered Massasauga Rattlesnake population in New York State. MS thesis, State University of New York, Syracuse.

Johnson, B. D., Gibbs, J. P., Shoemaker, K. T. & Cohen, J. B. (2016). Demography of a Small and Isolated Population of Eastern Massasauga Rattlesnakes (*Sistrurus catenatus*) Threatened by Vegetative Succession. *Journal of Herpetology,* **50,** 534-540.

Jones, P. C., King, R. B., Bailey, R. L., Bieser, N. D., Bissell, K., Campa, H., Crabill, T., Cross, M. D., Degregorio, B. A., Dreslik, M. J., Durbian, F. E., Harvey, D. S., Hecht, S. E., Jellen, B. C., Johnson, G., Kingsbury, B. A., Kowalski, M. J., Lee, J., Manning, J. V., Moore, J. A., Oakes, J., Phillips, C. A., Prior, K. A., Refsnider, J. M., Rouse, J. D., Sage, J. R., Seigel, R. A., Shepard, D. B., Smith, C. S., Vandewalle, T. J., Weatherhead, P. J. & Yagi, A. (2012). Range-wide analysis of eastern massasauga survivorship. *Journal of Wildlife Management,* **76,** 1576-1586.

Jones, P. C., King, R. B. & Sutton, S. (2017). Demographic analysis of imperiled Eastern Massasaugas (*Sistrurus catenatus catenatus*). *Journal of Herpetology*, **51,** 383-387.

King, R. B., Stanford, K. M., Jones, P. C. & Bekker, K. (2016). Size matters: individual variation in ectotherm growth and asymptotic size. *Plos One,* **11,** e0146299. doi:10.1371/journal.pone.0146299.

Kissner, K. J. & Weatherhead, P. J. (2005). Phenotypic effects on survival of neonatal northern watersnakes *Nerodia sipedon*. *Journal of Animal Ecology,* **74,** 259-265.

Koons, D. N., Birkhead, R. D., Boback, S. M., Williams, M. I. & Greene, M. P. (2009). The effect of body size on cottonmouth (*Agkistrodon piscivorus*) survival, recapture probability, and behavior in an Alabama swamp. *Herpetological Conservation and Biology,* **4,** 221-235.

Larsen, K. W. & Gregory, P. T. (1989). Population-size and survivorship of the common garter snake, *Thamnophis sirtalis*, near the northern limit of its distribution. *Holarctic Ecology,* **12,** 81-86.

Lelievre, H., Rivalan, P., Delmas, V., Ballouard, J. M., Bonnet, X., Blouin-Demers, G. & Lourdais, O. (2013). The thermoregulatory strategy of two sympatric colubrid snakes affects their demography. *Population Ecology,* **55,** 585-593.

Lind, A. J., Welsh, H. H. & Tallmon, D. A. (2005). Garter snake population dynamics from a 16-year study: Considerations for ecological monitoring. *Ecological Applications,* **15,** 294-303.

Luiselli, L., Capula, M. & Shine, R. (1997). Food habits, growth rates, and reproductive biology of grass snakes, *Natrix natrix* (Colubridae) in the Italian Alps. *Journal of Zoology,* **241,** 371-380.

Luiselli, L., Madsen, T., Capizzi, D., Rugiero, L., Pacini, N. & Capula, M. (2011). Long-term population dynamics in a Mediterranean aquatic snake. *Ecological Research,* **26,** 745-753.

Lyet, A., Cheylan, M., Prodon, R. & Besnard, A. (2009). Prescribed fire and conservation of a threatened mountain grassland specialist: a capture-recapture study on the Orsini's viper in the French Alps. *Animal Conservation,* **12,** 238-248.

Madsen, T. (1983). Growth-rates, maturation and sexual size dimorphism in a population of grass snakes, *Natrix natrix*, in southern Sweden. *Oikos,* **40,** 277-282.

Madsen, T. & Shine, R. (2000). Silver spoons and snake body sizes: prey availability early in life influences long-term growth rates of free-ranging pythons. *Journal of Animal Ecology,* **69,** 952-958.

Madsen, T., Ujvari, B., Shine, R. & Olsson, M. (2006). Rain, rats and pythons: Climate-driven population dynamics of predators and prey in tropical Australia. *Austral Ecology,* **31,** 30-37.

Maritz, B. (2011). Ecology of the Namaqua Dwarf Adder, *Bitis schneideri*. PhD thesis, University of the Witwatersrand,, Johannesburg.

Maritz, B. & Alexander, G. J. (2012). Population density and survival estimates of the African viperid, *Bitis schneideri*. *Herpetologica,* **68,** 195-202.

Marques, O. A. V., Kasperoviczus, K. & Almeida-Santos, S. M. (2013). Reproductive ecology of the threatened pitviper *Bothrops insularis* from Queimada Grande Island, Southeast Brazil. *Journal of Herpetology,* **47,** 393-399.

Masunaga, G. & Ota, H. (2003). Growth and reproduction of the sea snake, *Emydocephalus ijimae*, in the central Ryukyus, Japan: a mark and recapture study. *Zoological Science,* **20,** 461-470.

Miller, D. A., Clark, W. R., Arnold, S. J. & Bronikowski, A. M. (2011). Stochastic population dynamics in populations of western terrestrial garter snakes with divergent life histories. *Ecology,* **92,** 1658-1671.

Naulleau, G. & Bonnet, X. (1995). Reproductive ecology, body fat reserves and foraging mode in females of two contrasted snake species: *Vipera aspis* (terrestrial, viviparous) and *Elaphe longissima* (semi-arboreal oviparous). *Amphibia-Reptilia,* **16,** 37-46.

Nishimura, M. & Kamura, T. (1994). Survival rate and sex-ratio in habu, *Trimeresurus flavoviridis* (Viperidae), on the subtropical Okinawa island, Japan. *Researches on Population Ecology,* **36,** 115-120.

Olson, Z. H., MacGowan, B. J., Hamilton, M. T., Currylow, A. F. T. & Williams, R. N. (2015). Survival of Timber Rattlesnakes (*Crotalus horridus*): investigating individual, environmental, and ecological effects. *Herpetologica,* **71,** 274-279.

Parker, W. S. & Brown, W. S. (1980). Comparative ecology of two colubrid snakes, *Masticophis t. taeniatus* and *Pituophis melanoleucus deserticola*, in Northern Utah. *Milwaukee Public Museum Publications in Biology and Geology,* **7,** 1-104.

Phillips, B. L., Greenlees, M. J., Brown, G. P. & Shine, R. (2010). Predator behaviour and morphology mediates the impact of an invasive species: cane toads and death adders in Australia. *Animal Conservation,* **13,** 53-59.

Prival, D. B. & Schroff, M. J. (2012). A 13-year study of a northern population of twin-spotted rattlesnakes (*Crotalus pricei*): growth, reproduction, survival, and conservation. *Herpetological Monographs,* **26,** 1-18.

Riedle, J. D. (2014). Demography of an urban population of ring-necked snakes (*Diadophis punctatus*) in Missouri. *Herpetological Conservation and Biology,* **9,** 278-284.

Roe, J. H., Attum, O. & Kingsbury, B. A. (2013). Vital rates and population demographics in declining and stable watersnake populations. *Herpetological Conservation and Biology,* **8,** 591-601.

Rose, F. L., Simpson, T. R., Ott, J. R. & Manning, R. W. (2010). Use of space by western cottonmouths (*Agkistrodon piscivorus*) inhabiting a variable-flow stream. *Southwestern Naturalist,* **55,** 160-166.

Rose, F. L., Simpson, T. R., Ott, J. R., Manning, R. W. & Martin, J. (2010). Survival of western cottonmouths (*Agkistrodon piscivorus leucostoma*) in a pulsing environment. *Southwestern Naturalist,* **55,** 11-15.

Rossman, D. A., Ford, N. B. & Seigel, R. A. (1996). *The Garter Snakes Evolution and Ecology,* University of Oklahoma Press, Norman.

Sacerdote-Velat, A. B., Earnhardt, J. M., Mulkerin, D., Boehm, D. & Glowacki, G. (2014). Evaluation of headstarting and release techniques for population augmentation and reintroduction of the smooth green snake. *Animal Conservation,* **17,** 65-73.

Seigel, R. A. (1986). Ecology and conservation of an endangered rattlesnake, *Sistrurus catenatus*, in Missouri, USA. *Biological Conservation,* **35,** 333-346.

Sewell, D., Baker, J. M. R. & Griffiths, R. A. (2015). Population dynamics of grass snakes (*Natrix natrix*) at a site restored for amphibian reintroduction. *Herpetological Journal,* **25,** 155-161.

Shine, R. (1977). Reproduction in Australian elapid snakes 2. Female reproductive-cycles. *Australian Journal of Zoology,* **25,** 655-666.

Shine, R. (1984). Reproductive-biology and food-habits of the Australian elapid snakes of the genus *Cryptophis*. *Journal of Herpetology,* **18,** 33-39.

Shine, R., Brischoux, F. & Pile, A. J. (2010). A seasnake's colour affects its susceptibility to algal fouling. *Proceedings of the Royal Society B-Biological Sciences,* **277,** 2459-2464.

Shine, R., Shine, T. & Shine, B. (2003). Intraspecific habitat partitioning by the sea snake *Emydocephalus annulatus* (Serpentes, Hydrophiidae): the effects of sex, body size, and colour pattern. *Biological Journal of the Linnean Society,* **80,** 1-10.

Sperry, J. H. & Weatherhead, P. J. (2008). Prey-mediated effects of drought on condition and survival of a terrestrial snake. *Ecology,* **89,** 2770-2776.

Sperry, J. H. & Weatherhead, P. J. (2009). Sex differences in behavior associated with sex-biased mortality in an oviparous snake species. *Oikos,* **118,** 627-633.

Stanford, K. M. (2012). Spatial and temporal variation in demographic parameters of the Lake Erie Watersnake (*Nerodia sipedon insularum*). PhD thesis, Northern Illinois University, DeKalb, IL.

Stanford, K. M. & King, R. B. (2004). Growth, survival, and reproduction in a Northern Illinois population of the plains gartersnake, *Thamnophis radix*. *Copeia***,** 465-478.

Ujvari, B., Brown, G., Shine, R. & Madsen, T. (2016). Floods and famine: climate-induced collapse of a tropical predator-prey community. *Functional Ecology,* **30,** 453-458.

Waldron, J. L., Welch, S. M., Bennett, S. H., Kalinowsky, W. G. & Mousseau, T. A. (2013). Life history constraints contribute to the vulnerability of a declining North American rattlesnake. *Biological Conservation,* **159,** 530-538.

Weatherhead, P. J., Barry, F. E., Brown, G. P. & Forbes, M. R. L. (1995). Sex-ratios, mating-behavior and sexual size dimorphism of the northern water snake, *Nerodia sipedon*. *Behavioral Ecology and Sociobiology,* **36,** 301-311.

Webb, J. K., Brook, B. W. & Shine, R. (2002). What makes a species vulnerable to extinction? Comparative life-history traits of two sympatric snakes. *Ecological Research,* **17,** 59-67.

Webb, J. K., Christian, K. A. & Fisher, P. (2002). Fast growth and early maturation in a viviparous sit-and-wait predator, the northern death adder (*Acanthophis praelongus*), from tropical Australia. *Journal of Herpetology,* **36,** 505-509.

Webb, J. K. & Shine, R. (2008). Differential effects of an intense wildfire on survival of sympatric snakes. *Journal of Wildlife Management,* **72,** 1394-1398.

Whiting, M. J., Dixon, J. R., Greene, B. D., Mueller, J. M., Thornton, O. W., Hatfield, J. S., Nichols, J. D. & Hines, J. E. (2008). Population dynamics of the Concho Water Snake in rivers and reservoirs. *Copeia***,** **2008,** 438-445.

Williams, K. E., Hodges, K. E. & Bishop, C. A. (2014). Phenology and demography of great basin gophersnakes (*Pituophis catenifer deserticola*) at the northern edge of their range. *Herpetological Conservation and Biology,* **9,** 246-256.

Willson, J. D. & Winne, C. T. (2016). Evaluating the functional importance of secretive species: A case study of aquatic snake predators in isolated wetlands. *Journal of Zoology,* **298,** 266-273.

Willson, J. D., Winne, C. T. & Todd, B. D. (2011). Ecological and methodological factors affecting detectability and population estimation in elusive species. *Journal of Wildlife Management,* **75,** 36-45.

Winne, C. T., Willson, J. D. & Gibbons, J. W. (2006). Income breeding allows an aquatic snake *Seminatrix pygaea* to reproduce normally following prolonged drought-induced aestivation. *Journal of Animal Ecology,* **75,** 1352-1360.

Wylie, G. D., Casazza, M. L., Gregory, C. J. & Halstead, B. J. (2010). Abundance and Sexual Size Dimorphism of the Giant Gartersnake (*Thamnophis gigas*) in the Sacramento Valley of California. *Journal of Herpetology,* **44,** 94-103.

Zuffi, M. A. L., Fornasiero, S. & Bonnet, X. (2007). Geographic variation in reproductive output of female European whip snakes (*Hierophis viridiflavus*). *Herpetological Journal,* **17,** 219-224.
